# Pepper Leaf Extracts Alleviate HFD-Induced Metabolic Disorders via Microbiota-Driven Intestinal Barrier Repair and Bile Acid Reprogramming

**DOI:** 10.3390/nu18071105

**Published:** 2026-03-30

**Authors:** Ning Xu, Suxia Shen, Zhaotian Yang, Lin Zeng, Haifeng Zhang, Xiaojun Liao, Yan Zhang

**Affiliations:** 1 College of Food Science and Nutritional Engineering, China Agricultural University, Beijing 100083, China; 2 Sanya Institute of China Agricultural University, Sanya 572025, China; 3 Frontier Technology Research Institute of China Agricultural University in Shenzhen, Shenzhen 518000, China; 4 BGI Precision Nutrition (Shenzhen) Technology Co., Ltd., Shenzhen 518083, China

**Keywords:** pepper leaf extracts, fat accumulation, intestinal barrier, bile acid metabolism

## Abstract

**Background**: Obesity and its related metabolic complications, including non-alcoholic fatty liver disease (NAFLD) and insulin resistance, constitute an escalating global public health challenge, with high-fat diet (HFD) exposure recognized as a primary etiological driver. This study aimed to systematically evaluate the therapeutic effects of pepper leaf extracts (PLE), spinach extracts (SE), and obeticholic acid (OCA) on HFD-induced metabolic dysfunction in mice. **Methods**: Integrated phenotypic, histopathological, gut microbial, bile acid, and metabolomic analyses were applied to evaluate the intervention effects. **Results**: Our results demonstrated that 16-week dietary intervention with PLE, SE, or OCA all effectively mitigated HFD-induced obesity, pathological adipose remodeling, hepatic steatosis, systemic insulin resistance, and intestinal barrier dysfunction. Mechanistically, PLE effectively restored intestinal barrier integrity and reshaped the dysbiotic gut microbiota, with a marked enrichment of beneficial bacterial taxa closely linked to intestinal barrier maintenance, and normalized the disrupted cecal bile acid profile in HFD-fed mice. Furthermore, untargeted metabolomic analysis revealed that PLE reprogrammed disordered systemic metabolism, with significant modulation of key pathways involved in bile acid homeostasis, amino acid metabolism, and energy metabolism. **Conclusions**: In summary, this study provides evidence that PLE effectively attenuates HFD-induced metabolic disorders through modulation of the gut microbiota–bile acid–metabolome axis and restoration of intestinal barrier integrity. The superior therapeutic efficacy of PLE compared with SE and OCA, coupled with its favorable safety profile, positions PLE as a promising novel natural candidate for the prevention and treatment of obesity and its associated metabolic complications.

## 1. Introduction

The surging consumption of ultra-processed foods characterized by high fat and high sugar is a major driver of the global pandemic of metabolic diseases, including obesity, type 2 diabetes, and non-alcoholic fatty liver disease (NAFLD) [1,2]. Statistics show that more than 2.5 billion adults worldwide are overweight, among whom over 890 million suffer from obesity, and deaths directly or indirectly caused by metabolic diseases account for approximately 28% of the global annual total deaths [3]. As a core environmental risk factor, a high-fat diet (HFD) profoundly disrupts systemic metabolic homeostasis through multiple interconnected pathways, including perturbing the gut microecology, impairing intestinal barrier function, disrupting bile acid homeostasis, and dysregulating lipid and glucose metabolism [4,5].

One of the core driving factors of HFD-induced metabolic disorders lies in the imbalance of the gut microecology. Specifically, it is manifested by decreased microbial α-diversity, increased abundance of opportunistic pathogens, and reduced abundance of short-chain fatty acid (SCFA)-producing beneficial bacteria [6]. This gut microbiota dysbiosis is the initiating link of subsequent pathological changes: on the one hand, it directly impairs the structure and function of the intestinal barrier, leading to the downregulated expression of tight junction proteins (Occludin, Claudin, ZO-1) and thinning of the mucus layer [7,8]. This allows intestinal-derived endotoxins, such as lipopolysaccharide (LPS), to translocate into the systemic circulation, triggering “metabolic endotoxemia” and chronic low-grade inflammation, which in turn exacerbate hepatic steatosis and insulin resistance [9]. On the other hand, the dysregulated microbiota fails to normally catalyze the biotransformation of bile acids, resulting in disorders of the bile acid profile. Bile acids are not only essential for lipid digestion and absorption but also key signaling molecules that precisely regulate their own synthesis as well as systemic lipid and glucose metabolism through activating nuclear receptors such as farnesoid X receptor (FXR) [10]. Therefore, the homeostatic imbalance of the “gut microbiota–bile acid axis” constitutes a critical bridge linking HFD to host metabolic dysfunction.

Current intervention strategies for metabolic syndrome mainly include lifestyle interventions, drug therapy, and bariatric surgery [11]. However, due to poor long-term patient compliance, the effects of lifestyle interventions are often difficult to maintain. Although targeted drugs represented by obeticholic acid (OCA) can effectively activate FXR to regulate metabolism, their application is often accompanied by adverse reactions such as pruritus and dyslipidemia, and their efficacy is highly dependent on individual genetic background [12]. These limitations restrict their universality in the general population, especially in sub-healthy individuals or those with mild metabolic abnormalities. In view of this, exploring safe, effective, and easy-to-apply long-term interventions, especially natural bioactive compounds derived from daily diets, has become a research focus in the field of prevention and management of metabolic diseases [13].

Green vegetables are an important part of the daily diet and are recognized for their diverse health-promoting properties, which are associated with their abundant bioactive components. As a naturally occurring pigment widely present in plants and algae, chlorophyll has been extensively confirmed by numerous studies to possess various bioactivities, such as antioxidant, anti-inflammatory, toxin-binding and excretion-promoting effects. Pepper (*Capsicum annuum* L.) leaves have a history of traditional dietary use and contain bioactive compounds including phenols, flavonoids, and carotenoids, positioning them as potential functional food ingredients [14,15]. Spinach (*Spinacia oleracea* L.) is a green vegetable widely consumed across the globe and is recognized for its abundance of flavonoids, polyphenols, and carotenoids [16]. Both are rich in chlorophyll, making them ideal raw materials for chlorophyll extraction. Given the core role of the gut microbiota–bile acid axis in HFD-induced metabolic disorders, and the fact that chlorophyll and its derivatives (e.g., pheophytin) have been reported to regulate the gut microbiota and inhibit fatty acid synthase activity, we hypothesize that chlorophyll-rich plant extracts, such as pepper leaf extracts (PLE) and spinach extracts (SE), may exert the potential to improve metabolic disorders by regulating this axis [17,18].

This study adopted an HFD-induced obese mouse model and used multi-omics techniques (phenotypic analysis, histopathological evaluation, 16S rRNA gene sequencing, targeted bile acid profiling, and untargeted liver metabolomics) to systematically explore the protective effects and potential mechanisms of PLE against HFD-induced metabolic disorders. To comprehensively evaluate the ameliorative effects of PLE, a dual-control design was employed, comprising SE as a vegetable-derived control and obeticholic acid (OCA), a clinically investigated FXR agonist, as a positive pharmacological control. The objectives of this study were to clarify the therapeutic efficacy differences among PLE, SE and OCA in improving HFD-induced metabolic disorders, explore the potential regulatory pathways of PLE (focusing on the gut microbiota, bile acid metabolism and intestinal barrier function), and preliminarily evaluate its safety. This study aims to provide solid scientific evidence for developing PLE as an effective and safe dietary intervention strategy for the prevention and management of obesity and its associated metabolic complications.

## 2. Materials and Methods

### 2.1. Materials

Fresh pepper leaves were harvested from the pepper cultivar *Honglong 23* in Bayannur City, Inner Mongolia Autonomous Region, China. Fresh spinach cultivar *Hubo 1* was purchased from a local market near China Agricultural University (Beijing, China). Obeticholic acid (OCA, purity ≥ 98%) was obtained from Yuanye Bio-Technology Co., Ltd. (Shanghai, China). The control diet (XT304), high-fat diet (HFD, XT310), and customized experimental diets were all purchased from Xietong Bioengineering Co., Ltd. (Nanjing, China). The ingredient and energy supply ratio of the two diets are provided in Supplementary Table S1. Hematoxylin-eosin (H&E) staining kit, Oil Red O staining kit, insulin assay kit, liver total cholesterol (TC) and triglyceride (TG) assay kits were purchased from Solarbio Science & Technology Co., Ltd. (Beijing, China). Lipopolysaccharide (LPS) content assay kit and diamine oxidase (DAO) activity assay kit were also provided by Solarbio Science & Technology Co., Ltd. (Beijing, China). Human insulin injection was obtained from Novo Nordisk (China) Pharmaceutical Co., Ltd. (Beijing, China), at a specification of 300 U/3 mL per pen. Liquid chromatography-mass spectrometry (LC-MS) grade methanol, formic acid, isopropanol, and acetonitrile were purchased from Thermo Fisher Scientific (China) Co., Ltd. (Beijing, China). The purified water used in all experiments of this study was purchased from Wahaha Group Co., Ltd. (Hangzhou, China).

### 2.2. Preparation of Pepper Leaf Extracts and Spinach Extracts

Pepper leaf extracts (PLE) and spinach extracts (SE) were prepared separately using an identical ethanol extraction and enrichment process, with the only difference in the pre-treatment step as specified below.

For PLE preparation: fresh pepper leaves were mixed with 95% ethanol at a solid–liquid ratio of 1:3 (*w*/*v*), and homogenized in a blender. The resulting homogenate was aliquoted into 250 mL centrifuge bottles and centrifuged at 8000× *g* for 10 min at 4 °C. The supernatant was collected and concentrated to approximately one-fourth of the original volume via rotary evaporation (RE-3000A, Shanghai Yarong Biochemical Instrument Factory, Shanghai, China) at 36 °C, followed by physical sedimentation in a 4 °C refrigerator (Haier, Qingdao, China) for 12 h. Subsequently, the upper liquid layer was discarded, and the dark green thick slurry at the bottom was collected as the final PLE.

SE was prepared using exactly the same procedure described above, except for the pre-treatment: fresh spinach was destemmed before homogenization, and subjected to the same centrifugation, rotary evaporation, low-temperature sedimentation, and collection steps to obtain the final SE. The ingredient lists of the two extracts are included in Supplementary Table S2. PLE and SE were added to the basal diets XT304 and XT310 by the manufacturer to formulate the experimental diets for the CON_PLE, HFD_PLE, and HFD_SE groups (Table 1).

### 2.3. Experimental Animals

All animal experiments were conducted under standard laboratory conditions and approved by the Animal Ethics Committee of China Agricultural University. Thirty-six 5-week-old male C57BL/6J mice were purchased from SPF (Beijing) Biotechnology Co., Ltd. (Beijing, China). All mice were housed in a specific pathogen-free (SPF) environment with a relative humidity of 55 ± 5%, an ambient temperature of 23 ± 2 °C, and a 12 h light/12-h dark cycle, with free access to diet and water throughout the experimental period.

After a 2-week acclimation period, healthy mice were randomly divided into 6 groups (*n* = 6 per group) using a random number table method. The detailed grouping, dietary formula, and gavage intervention protocols are summarized in Table 1. Briefly, during the 16-week intervention period: mice in the control group (CON) were fed a basal control diet (XT304); mice in the control + pepper leaf extracts group (CON_PLE) were fed a basal control diet supplemented with PLE (XT304 + 2%PLE); mice in the high-fat diet group (HFD) were fed a pure high-fat diet (XT310); mice in the high-fat diet + PLE group (HFD_PLE) were fed a high-fat diet supplemented with PLE (XT310 + 2%PLE); mice in the high-fat diet + spinach extracts group (HFD_SE) were fed a high-fat diet supplemented with SE (XT310 + 1.98%SE); mice in the high-fat diet + obeticholic acid group (HFD_OCA) were fed a pure high-fat diet (XT310).

All gavage interventions were performed once daily at a fixed time, with a uniform gavage volume of 10 mL/kg body weight to eliminate operational bias and gavage stress interference. Mice in all groups except the HFD_OCA group were gavaged with an equal volume of 0.5% sodium carboxymethyl cellulose (CMC-Na; Solarbio, Beijing, China) solution. Mice in the HFD_OCA group were orally gavaged with 10 mg/kg body weight per day of obeticholic acid (OCA) suspended in 0.5% CMC-Na solution, which served as the positive control for metabolic regulation.

The supplemental dose of PLE and SE in the diet was set in strict accordance with the Dietary Guidelines for Chinese Residents. Specifically, the recommended daily intake of fresh vegetables for healthy adults is 300–500 g, and the final supplemental dose in mouse feed was determined based on the chlorophyll content in 500 g of fresh spinach, with the equivalent dose converted via the body surface area method. Precisely weighed PLE and SE were repeatedly dissolved in anhydrous ethanol, centrifuged, and the supernatant was collected until the samples appeared gray. After gradient dilution, the absorbance of the extracts was measured at 645 nm and 663 nm, respectively. The total chlorophyll concentration (mg/L) was calculated using the following equation: C = 8.04 × A_663_ + 20.29 × A_645_.

The HFD_SE group was set to serve as a validated experimental reference consistent with our previous research work, to quantitatively evaluate the intervention efficacy of PLE on high-fat diet-induced metabolic disorders.

All animal experiments were conducted in strict accordance with the guidelines for the care and use of laboratory animals issued by the Animal Ethics Committee of China Agricultural University. The experimental protocol was reviewed and approved by the above committee (approval number: AW61214202-5-01, 16 December 2024).

### 2.4. Physiologic Measurements and Final Collection of Samples

Body weight and food intake of the mice were monitored consecutively for 16 weeks, with weekly documentation. Total energy intake was quantified across the entire 16-week intervention period. Total feed consumption per cage was measured, divided by the number of mice per cage to obtain the mean feed intake per animal, and then multiplied by the defined gross energy density of the corresponding diet. The inclusion rates of pepper leaf extracts (PLE) and spinach extracts (SE) in the modified diets were maintained at 1.98–2%, such that macronutrient composition and energy density differed only marginally and non-significantly between the modified diets and the high-fat diet (Supplementary Table S1). Upon completion of the experimental period, blood samples were procured via aseptic orbital venous plexus puncture. Following gentle inversion to ensure homogeneous mixing, the blood samples were centrifuged at 3000 rpm and 4 °C for 15 min. The resulting supernatant was promptly flash-frozen in liquid nitrogen for 30 s prior to long-term preservation at −80 °C until subsequent biochemical analyses. Mice were humanely euthanized by cervical dislocation, following which liver, epididymal white adipose tissue (eWAT), inguinal white adipose tissue (iWAT), perirenal white adipose tissue (pWAT), and scapular brown adipose tissue (sBAT), as well as jejunal and ileal tissues and cecal contents, were harvested aseptically. The isolated tissues were blotted gently to remove excess moisture using filter paper, weighed accurately, and then immediately transferred to liquid nitrogen for rapid cryopreservation, prior to being stored at −80 °C for downstream experimental assays. Blood glucose was measured using a portable blood glucose meter (Accu-Chek Performa, Roche, Basel, Switzerland). Serum insulin levels were determined using an insulin assay kit (Solarbio Science & Technology Co., Ltd., Beijing, China). Liver total cholesterol (TC) and triglyceride (TG) levels were determined using a liver TC and TG assay kit (Solarbio Science & Technology Co., Ltd., Beijing, China).

### 2.5. Histological Analysis

Adipose tissue, jejunum, and ileum were fully immersed in 4% paraformaldehyde for 48 h of fixation, followed by gradient dehydration, paraffin embedding, and sectioning into 4-μm-thick serial sections.

#### 2.5.1. H&E Staining

Prepared paraffin sections of adipose and liver tissues were stained with hematoxylin and eosin (H&E) in strict accordance with protocols provided by Servicebio (Wuhan, China). Following staining, the H&E-stained adipose tissue sections were examined under a conventional light microscope for histological evaluation.

#### 2.5.2. Oil Red O Staining

Prepared frozen sections of liver tissue were stained with Oil Red O in strict accordance with protocols provided by Servicebio (Wuhan, China). Following staining, the Oil Red O-stained liver tissue sections were examined under a conventional light microscope for lipid accumulation evaluation.

#### 2.5.3. Periodic Acid Schiff (PAS) Glycogen Staining

Firstly, paraffin sections were deparaffinized by sequential immersion in anhydrous ethanol, 95% ethanol, 90% ethanol, 80% ethanol, and 70% ethanol (5 min per step), followed by rinsing with distilled water for rehydration. Following deparaffinization and rehydration, the slices were treated with a 0.5% periodic acid aqueous solution for 10 min, rinsed with running water, and washed twice with distilled water. Subsequently, the slices were immersed in Schiff’s reagent for 30 min of staining in the dark, then rinsed with running water for 10 min. Cell nuclei were counterstained with hematoxylin for 2 min, washed with distilled water, and differentiated in a 1% hydrochloric acid-ethanol solution for several seconds. After rinsing with distilled water for a few minutes, the slices were blued. Subsequent to counterstaining, the slices were sequentially subjected to dehydration and clearing in 95% ethanol, anhydrous ethanol, and xylene (10 min per step). Finally, the dried slices were mounted with neutral balsam, examined under a microscope, and images were collected and analyzed using ImageJ (version 1.80) software.

#### 2.5.4. Immunofluorescence

Paraffin-embedded jejunal and ileal sections were first baked at 60 °C to facilitate deparaffinization. Subsequently, the sections were subjected to deparaffinization, permeabilization, and antigen retrieval to expose epitopes. To mitigate non-specific binding, the sections were blocked with 5% goat serum (diluted in PBS), followed by overnight incubation at 4 °C with ZO-1, Occludin, and Claudin primary antibodies (1:100 dilution; Servicebio, Wuhan, China). On the following day, the sections were incubated with fluorescently conjugated secondary antibodies (1:200 dilution; Servicebio, Wuhan, China) at room temperature for 3 h. Nuclei were counterstained with DAPI (Thermo Fisher Scientific, Waltham, MA, USA), and fluorescence imaging was performed using a Leica DM6B fluorescence microscope to assess protein expression patterns and subcellular localization.

### 2.6. 16S rDNA Sequence Analysis

Total microbial genomic DNA was extracted from the cecal contents of mice using the FastPure Stool DNA Isolation Kit (MJYH, Shanghai, China) following the manufacturer’s instructions. The integrity of the extracted DNA was verified via 1% agarose gel electrophoresis, and the concentration and purity were quantified using a NanoDrop 2000 spectrophotometer (Thermo Fisher Scientific, Waltham, MA, USA).

The V3-V4 hypervariable region of the bacterial 16S rRNA gene was amplified via polymerase chain reaction (PCR) using the barcoded universal primer pair 338F (5′-ACTCCTACGGGAGGCAGCAG-3′) and 806R (5′-GGACTACHVGGGTWTCTAAT-3′), with qualified genomic DNA as the template. PCR amplification was performed in a total reaction volume of 20 μL, containing 4 μL of 5× TransStart FastPfu Buffer, 2 μL of 2.5 mM dNTPs, 0.8 μL of each forward and reverse primer (5 μM), 0.4 μL of TransStart FastPfu DNA Polymerase (TransGen Biotech, Beijing, China), 10 ng of template DNA, and nuclease-free ddH_2_O to adjust the final volume to 20 μL. The amplification was conducted on an ABI GeneAmp^®^ 9700 thermal cycler (Applied Biosystems, Foster City, CA, USA) with the following program: initial denaturation at 95 °C for 3 min; 27 cycles of denaturation at 95 °C for 30 s, annealing at 55 °C for 30 s, and extension at 72 °C for 30 s; a final extension step at 72 °C for 10 min; the amplified products were stored at 4 °C until subsequent processing.

The PCR amplicons were separated via 2% agarose gel electrophoresis, purified using a PCR Clean-Up Kit (Yuhua, China), and accurately quantified with a Qubit 4.0 Fluorometer (Thermo Fisher Scientific, Waltham, MA, USA).

Sequencing libraries were constructed using the NEXTFLEX Rapid DNA-Seq Kit (PerkinElmer, Austin, TX, USA) following the manufacturer’s standard protocol, which included adapter ligation, magnetic bead-based removal of self-ligated fragments, library enrichment amplification, and final product purification. Qualified libraries were sequenced on an Illumina NextSeq 2000 platform (Illumina, San Diego, CA, USA).

Raw paired-end reads were quality-controlled with Fastp (trimming low-quality bases, filtering short/ambiguous reads, https://github.com/OpenGene/fastp, version 0.19.6, accessed on 1 March 2026) and assembled with FLASH (overlap ≥ 10 bp, mismatch ratio ≤ 0.2, https://ccb.jhu.edu/software/FLASH/, version 1.2.11, accessed on 1 March 2026). Samples were distinguished by barcodes/primers, and sequences were denoised into amplicon sequence variants (ASVs) using the DADA2 plugin in QIIME 2 (default parameters). Chloroplast/mitochondrial sequences were removed, and all samples were rarefied to 20,000 reads (Good’s coverage: 99.09%).

ASV taxonomic annotation was conducted against the Silva 138 database via the Naive Bayes classifier in QIIME 2. Alpha diversity indices were calculated with Mothur (http://www.mothur.org/wiki/Calculators, accessed on 1 March 2026) and compared using the Wilcoxon rank-sum test. Beta diversity was evaluated by Bray–Curtis-based PCoA, with group differences tested via PERMANOVA. Functional prediction of 16S rRNA genes was performed using PICRUSt2 (version 2.2.0).

### 2.7. Determination of Serum DAO and LPS Levels

Prepared serum samples were analyzed for diamine oxidase (DAO) and lipopolysaccharide (LPS) contents using commercial assay kits in strict accordance with protocols provided by Solarbio Science & Technology Co., Ltd. (Beijing, China).

### 2.8. Determination of Bile Acids in Cecal Contents

Preparation of standard and internal standard solutions was performed prior to sample analysis. Forty-seven bile acid standards were accurately weighed, and individual stock solutions were prepared using chromatographic-grade methanol. These stock solutions were subsequently mixed and serially diluted with 50% chromatographic-grade acetonitrile to generate working solutions of gradient concentrations. Isotope internal standards (CDCA-D4, CA-D4) were precisely weighed, dissolved in methanol to prepare stock solutions, and further diluted to final concentrations of 2000 ng/mL (internal standard 1) and 200 ng/mL (internal standard 2). Working standard solutions were mixed with internal standard 2 at a volume ratio of 1:1 to prepare linear solutions (L1–L12) for standard curve construction. All standard and internal standard solutions were stored at −20 °C in the dark prior to use to prevent degradation.

For sample pretreatment, 25 mg of cecal contents was accurately weighed into a sterile centrifuge tube and fully homogenized to ensure uniform sampling. Subsequently, 20 μL of internal standard 1 and 380 μL of chromatographic-grade extraction solution (methanol:water = 4:1, *v*/*v*) were added to the tube. The mixture was vortexed vigorously for 1 min to facilitate thorough mixing of the sample and extraction solution, followed by frozen grinding for 6 min (−10 °C, 50 Hz) and low-temperature ultrasonic extraction for 30 min (5 °C, 40 KHz). After extraction, the mixture was incubated at −20 °C for 30 min to promote protein precipitation. The mixture was then centrifuged at 13,000× *g* for 15 min at 4 °C, and 200 μL of the resulting supernatant was carefully collected. The supernatant was filtered through a 0.22 μm organic phase filter membrane to remove impurities and immediately placed on the instrument sample rack for subsequent detection to avoid solvent volatilization and component alteration.

Qualitative and quantitative analysis of bile acids was conducted using liquid chromatography-electrospray ionization-tandem mass spectrometry (LC-ESI-MS/MS, UHPLC-Qtrap) following preheating and calibration of the instrument to ensure operational stability. Chromatographic separation was achieved using an ExionLC AD system (AB Sciex, Framingham, MA, USA) equipped with a Waters BEH C18 column (150 × 2.1 mm, 1.7 μm; Waters, Milford, MA, USA), maintained at 35 °C. The injection volume was 1 μL, and the flow rate was set at 0.3 mL/min. The mobile phase consisted of 0.1% formic acid in water (phase A) and 0.1% formic acid in acetonitrile (phase B), both of chromatographic grade, with a gradient elution program employed to ensure effective separation of target bile acid components. Mass spectrometric detection was performed using an AB SCIEX QTRAP 6500+ mass spectrometer (AB Sciex, Framingham, MA, USA) in negative ion mode, with the following parameters: curtain gas (CUR) = 35, collision-activated dissociation (CAD) = Medium, ion spray voltage (IS) = −4500 V, temperature (TEM) = 550 °C, gas 1 (GS1) = 50, gas 2 (GS2) = 50. Multiple reaction monitoring (MRM) mode was utilized for quantitative detection, with pre-optimized parent and daughter ion pairs for each bile acid component to enhance detection specificity.

Ion fragments were automatically identified and integrated using AB Sciex OS software (version 4.0; AB Sciex, Framingham, MA, USA), with manual verification performed to correct false peaks and ensure accurate peak integration. A standard curve was constructed by plotting the ratio of the peak area of each bile acid analyte to that of the internal standard as the ordinate, against the concentration of the analyte as the abscissa. A correlation coefficient (R^2^) greater than 0.99 was required to validate the linearity of the standard curve. The concentration of each bile acid component in the cecal content samples was calculated by substituting the corresponding peak area ratio of the sample into the standard curve. Quality control (QC) samples were prepared by mixing equal volumes of all sample supernatants and detected intermittently (one QC sample per 10 test samples) to monitor the stability and accuracy of the detection method throughout the experiment.

### 2.9. Metabolomics Analysis

For serum metabolomic analysis, 100 μL serum aliquots were mixed with 400 μL acetonitrile-methanol (1:1, *v*/*v*) containing four internal standards (e.g., L-2-chloroalanine, 0.02 mg/mL). The mixtures were vortexed, subjected to low-temperature ultrasonic extraction (5 °C, 40 kHz, 30 min), incubated at −20 °C for protein precipitation, and centrifuged (13,000× *g*, 4 °C, 15 min). Supernatants were dried under nitrogen, reconstituted in 100 μL acetonitrile-water (1:1, *v*/*v*), re-sonicated, and re-centrifuged; the final supernatants were analyzed via UHPLC-Orbitrap Exploris 240 system (Thermo Fisher Scientific, Waltham, MA, USA).

Quality control (QC) samples were prepared by pooling equal volumes of all serum samples and injected intermittently to monitor analytical stability. Chromatographic separation was performed on an Acquity UHPLC HSS T3 column (100 mm × 2.1 mm, 1.8 μm; Waters, Milford, MA, USA) with mobile phases A (0.1% formic acid in water-acetonitrile (95:5, *v*/*v*)) and B (0.1% formic acid in acetonitrile-isopropanol-water (47.5:47.5:5, *v*/*v*/*v*)), at 0.40 mL/min and 40 °C with a default linear gradient program. Mass spectra were acquired in positive/negative ESI modes (*m*/*z* 70–1050) under optimized parameters, with full-scan high-resolution detection (120,000 resolution at *m*/*z* 200).

Raw data were processed via Progenesis QI software (version 2.4) for baseline correction, peak alignment, and integration. Metabolites were identified by matching MS/MS spectra against HMDB and METLIN databases (mass tolerance ± 5 ppm). Data preprocessing included filtering variables with >20% missing values, imputing remaining missing values with the minimum intensity, normalizing via global sum method, excluding variables with RSD > 30% in QC samples, and log10-transformation.

Orthogonal partial least squares discriminant analysis (OPLS-DA) was performed using R package ropls (version 1.6.2), with 7-fold leave-one-out cross-validation to evaluate model robustness (R^2^Y, Q^2^). Significantly differential metabolites were screened by VIP > 1 (from OPLS-DA) and *p* < 0.05 (two-tailed Student’s *t*-test).

Metabolic pathway annotation of differential metabolites was performed using the Kyoto Encyclopedia of Genes and Genomes (KEGG) database (https://www.kegg.jp/kegg/pathway.html, accessed on 7 September 2025). Pathway enrichment analysis was carried out using the scipy.stats package in Python (v3.9), with Fisher’s exact test (two-tailed) applied to identify biologically relevant pathways (adjusted *p* < 0.05 considered statistically significant).

### 2.10. Statistical Analysis

Statistical analysis was performed using GraphPad Prism 10.00 software (GraphPad Software, Boston, MA, USA). The data were expressed as mean ± standard deviation (SD). Differences between groups were analyzed using one-way analysis of variance (ANOVA) followed by Tukey’s multiple comparison test. Statistical significance was defined as *p* < 0.05.

## 3. Results

### 3.1. Pepper Leaf Extracts Alleviate Ectopic Fat Deposition, Hepatic Lipid Accumulation and Insulin Resistance in Obese Mice

**Figure 1:**
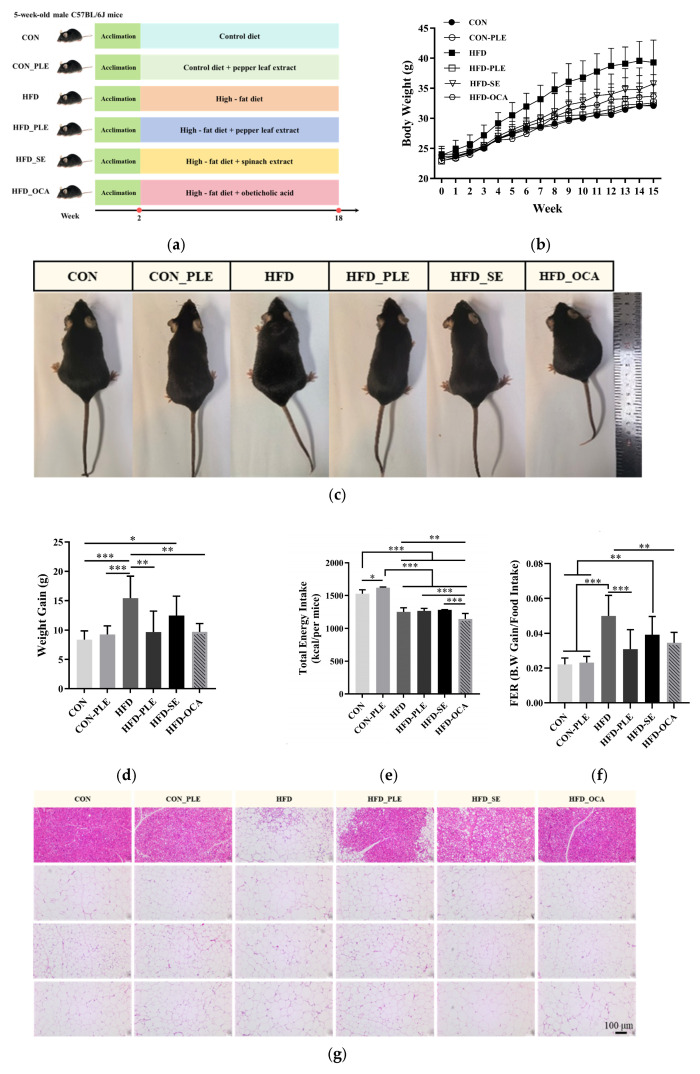

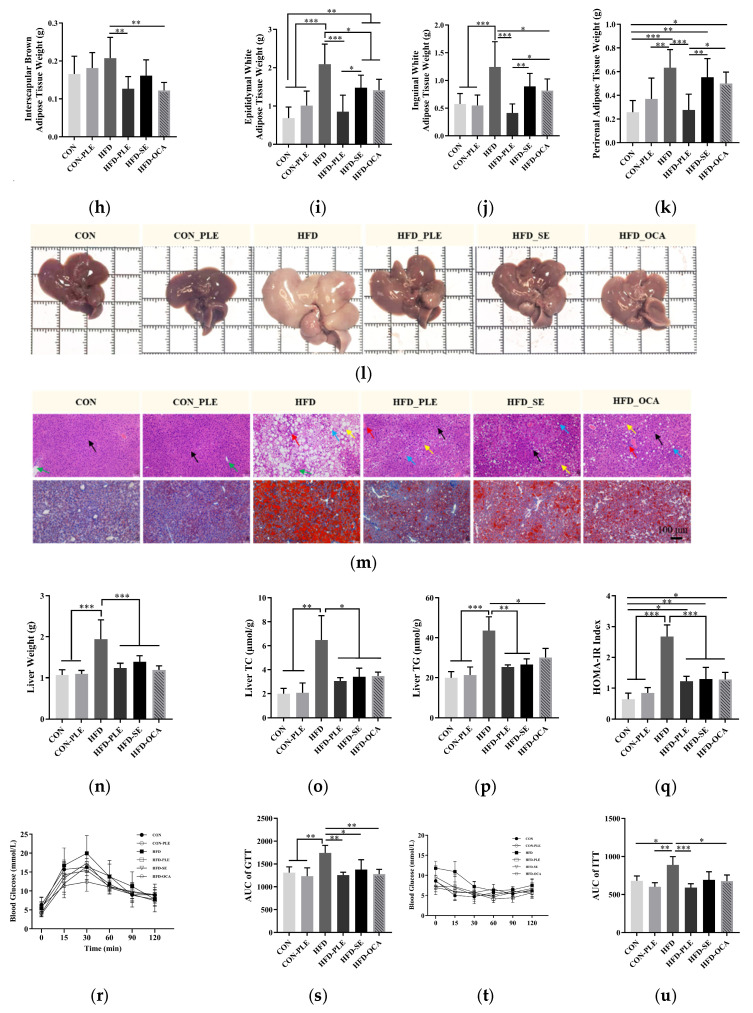
Pepper leaf extracts (PLE) ameliorated the growth phenotype, adipose accumulation, hepatic lipid metabolism and glucose homeostasis in mice fed with a high-fat diet. (**a**) The experimental plan of this study. (**b**) Mouse body weight. (**c**) Mouse appearance. (**d**) Weight gain. (**e**) Total energy intake. (**f**) Feeding efficiency ratio (FER). (**g**) H&E staining of various adipose tissues. (**h**) Interscapular brown adipose tissue weight. (**i**) Epididymal white adipose tissue weight. (**j**) Inguinal white adipose tissue weight. (**k**) Perirenal adipose tissue weight. (**l**) Liver appearance. (**m**) Liver H&E and Oil Red O staining (Black arrows indicate normal hepatocytes; green arrows indicate normal portal areas; blue arrows indicate hepatic steatosis/lipid droplets; yellow arrows indicate hepatocyte ballooning degeneration; red arrows indicate inflammatory cell infiltration). (**n**) Liver weight. (**o**) Liver TC content (total cholesterol content). (**p**) Liver TG content (triglyceride content). (**q**) HOMA-IR index (homeostatic model assessment for insulin resistance). (**r**) Glucose tolerance test (GTT). (**s**) AUC of GTT (area under the curve of glucose tolerance test). (**t**) Insulin tolerance test (ITT) Mice were injected intraperitoneally with insulin solution at a dosage of 0.75 U/kg body weight. (**u**) AUC of ITT (area under the curve of insulin tolerance test).* *p* < 0.05, ** *p* < 0.01, *** *p* < 0.001; *n* = 6. Scale bar: 100 μm.

Following 16 weeks of dietary intervention, the obese phenotypes of mice in the HFD_PLE, HFD_SE, and HFD_OCA groups were significantly ameliorated (Figure 1a,c). At the termination of the experiment, compared with the HFD group, body weight was reduced by 17.12%, 9.08%, and 14.32% in the HFD_PLE, HFD_SE, and HFD_OCA groups, respectively (Figure 1b). As illustrated in Figure 1d, mice in the HFD_PLE and HFD_OCA groups exhibited a marked reduction in body weight gain compared with the HFD group (*p* < 0.01), whereas a non-significant reduction in body weight was observed in the HFD_SE group (*p* > 0.05). Notably, no significant difference in energy intake was detected among the HFD_PLE, HFD_SE, and HFD groups (*p* > 0.05). In contrast, a significant difference in energy intake was identified between the HFD_OCA and HFD groups (*p* < 0.01) (Figure 1e). Furthermore, compared with the Food Efficiency Ratio (FER) of the HFD group, the FER was extremely significantly decreased in the HFD_PLE group (*p* < 0.001) and significantly decreased in the HFD_OCA group (*p* < 0.01) (Figure 1f).

Hematoxylin and eosin (H&E) staining demonstrated that, compared with the CON group, brown adipose tissue of HFD mice exhibited extensive whitening and severe vacuolar degeneration, with the disappearance of the typical multilocular lipid droplet structure and a significant attenuation of brown adipose tissue characteristics. Meanwhile, three types of white adipocytes (epididymal, inguinal, and perirenal adipose tissue) were significantly hypertrophied with increased cell diameter in HFD mice (Figure 1g). Quantitative analysis revealed that the relative weights of epididymal, inguinal, and perirenal white adipose tissue were significantly increased in HFD mice compared with the CON group (*p* < 0.001). Compared with the HFD group, the relative weight of brown adipose tissue was significantly decreased in the HFD_PLE group (*p* < 0.01), and the relative weights of epididymal, inguinal, and perirenal adipose tissue were extremely significantly reduced (*p* < 0.001). A significant reduction in the relative weight of epididymal adipose tissue was also observed in the HFD_SE group (*p* < 0.05). In the HFD_OCA group, the relative weight of brown adipose tissue was significantly decreased (*p* < 0.01), along with a significant reduction in the relative weights of epididymal and inguinal adipose tissue (*p* < 0.05) (Figure 1h–k).

In contrast to the livers of CON and CON_PLE mice, which displayed a ruddy color and tender texture, livers of HFD mice were significantly enlarged and presented a pale yellow to whitish appearance, indicating severe hepatic lipid deposition and steatosis. Compared with the HFD group, the degree of hepatic hypertrophy was significantly alleviated and liver coloration was notably improved in the HFD_PLE, HFD_SE, and HFD_OCA groups (Figure 1l).

Hepatic lobule structures were intact and distinct in CON and CON_PLE mice, with hepatic cords arranged in a regular radial pattern centered on the central vein. Hepatocytes were uniform in size (black arrows) with plump and abundant cytoplasm; portal areas exhibited a normal structure (green arrows), without obvious inflammatory cell infiltration or fibrotic changes. In HFD mice, hepatic lobule structures were severely obscure, and hepatic cords were disorganized. Hepatocytes showed severe steatosis (blue arrows), with lipid droplets predominantly large vacuolar and extensively fused into sheets; a large number of hepatocytes were abnormally enlarged, presenting obvious ballooning degeneration (yellow arrows). Multifocal inflammatory cell aggregates were observed within hepatic lobules (red arrows), with most areas occupied by lipid droplets. Early fibrotic lesions were detected around portal areas and central veins, extending into hepatic lobules and accompanied by obvious inflammatory cell infiltration (green arrows). In HFD_PLE mice, hepatic lobule structures were relatively distinct, with slightly disorganized hepatic cords and uneven hepatocyte sizes (black arrows). Only mild steatosis was observed (blue arrows), with lipid droplets mainly small vacuolar; occasional slight ballooning degeneration was noted in a very small number of hepatocytes (yellow arrows), and only mild lymphocyte infiltration was detected in portal areas (red arrows). Hepatic lobule structures were distinguishable in HFD_SE mice, with slightly disorganized hepatic cords and uneven hepatocyte sizes (black arrows). Occasional ballooning degeneration was observed, and hepatocyte steatosis was mild to moderate (blue arrows), with a lower proportion of large vacuolar steatosis than that in the HFD_OCA group (yellow arrows); the degree of inflammatory cell infiltration in portal areas was comparable to that in the HFD_OCA group (red arrows). In HFD_OCA mice, hepatic lobule structures were slightly disorganized, with uneven hepatocyte sizes (black arrows) and mild to moderate steatosis (blue arrows). The proportion of large vacuolar lipid droplets was increased, with scattered ballooning hepatocytes (yellow arrows); the number of inflammatory cells infiltrating portal areas was greater than that in the HFD_PLE group but significantly less than that in the HFD group. Only a small number of red lipid droplet signals were detected in livers of CON and CON_PLE mice. In contrast, extensive strong positive red staining was observed in livers of HFD mice, indicating massive abnormal hepatic lipid deposition. Compared with the HFD group, the area of red lipid droplets was significantly reduced in the HFD_PLE, HFD_SE, and HFD_OCA groups, with the smallest area in the HFD_PLE group, followed by the HFD_OCA group, and the largest in the HFD_SE group. These findings indicate that all three interventions can alleviate hepatic lipid deposition, with pepper leaf extract exerting the most prominent effect (Figure 1m).

Quantitative analysis of liver weight showed that liver weight in the HFD group was significantly higher than that in all other groups, with extremely significant differences (*p* < 0.001) (Figure 1n). Regarding hepatic total cholesterol (TC) content, compared with the HFD group, TC levels were extremely significantly decreased in the CON and CON_PLE groups (*p* < 0.01), and significantly reduced in the HFD_PLE, HFD_SE, and HFD_OCA groups (*p* < 0.05), with the lowest TC level observed in the HFD_PLE group (Figure 1o). For hepatic triglyceride (TG) content, compared with the HFD group, TG levels were extremely significantly decreased in the CON and CON_PLE groups (*p* < 0.001), significantly reduced in the HFD_PLE and HFD_SE groups (*p* < 0.01), and significantly lowered in the HFD_OCA group (*p* < 0.05), with the lowest TG level detected in the HFD_PLE group (Figure 1p).

Compared with the CON and CON_PLE groups, the area under the curve of glucose tolerance test (AUC of GTT) was significantly increased in HFD mice (*p* < 0.01, Figure 1s), indicating a significant impairment of glucose tolerance. The AUC of insulin tolerance test (ITT) was also significantly elevated in HFD mice (*p* < 0.05 or *p* < 0.01, Figure 1t), suggesting decreased insulin sensitivity. Meanwhile, the HOMA-IR index was extremely significantly increased in the HFD group (*p* < 0.001, Figure 1q), demonstrating that high-fat diet induced obvious insulin resistance in mice. Compared with the HFD group, the AUC of GTT was significantly decreased in the HFD_PLE and HFD_OCA groups (*p* < 0.01), and HFD_SE treatment also significantly improved glucose intolerance (*p* < 0.05). In terms of insulin sensitivity, HFD_PLE extremely significantly reversed the elevation of AUC of ITT (*p* < 0.001), and HFD_OCA significantly ameliorated insulin resistance (*p* < 0.05), whereas HFD_SE had no significant ameliorative effect (*p* > 0.05). Additionally, all three intervention strategies extremely significantly decreased the HOMA-IR index (*p* < 0.001).

### 3.2. Pepper Leaf Extracts Restore the Small Intestinal Barrier

**Figure 2:**
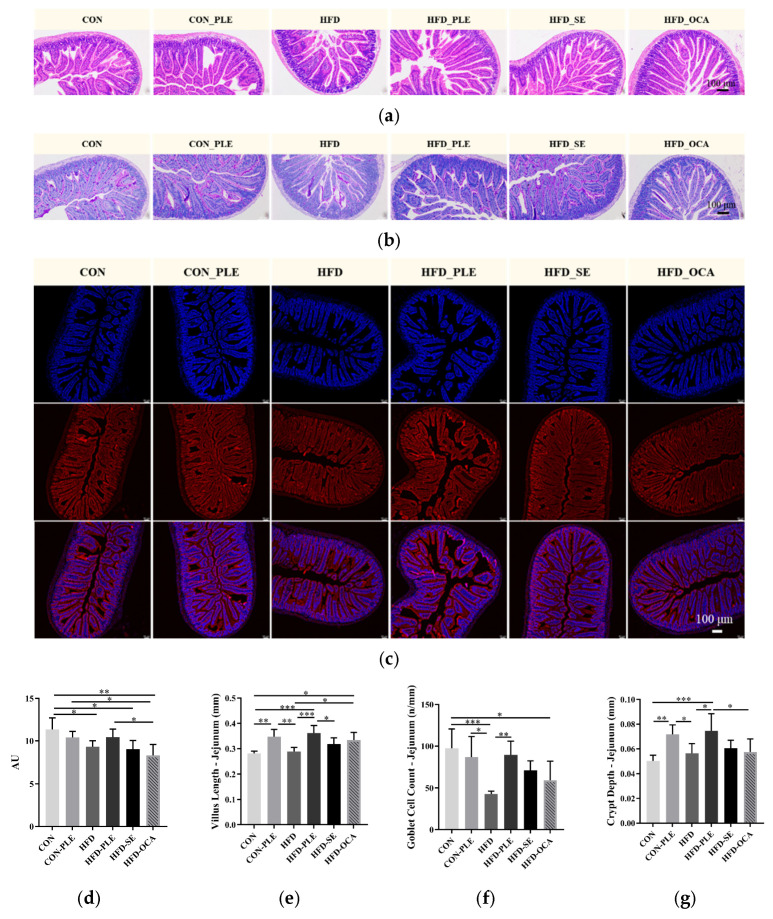
Pepper leaf extracts (PLE) improved intestinal barrier in jejunum. (**a**) Representative graph of jejunum H&E staining (100×). (**b**) Representative graph of jejunum PAS staining (100×). (**c**) Representative graph of jejunum Occludin immunofluorescence (Blue: DAPI (nucleus); red: Occludin; magenta: merged signals; 100×). (**d**) Immunofluorescence positive surface density of Occludin protein in jejunum. (**e**) Villus length. (**f**) Goblet cell count. (**g**) Crypt depth. The positive surface density of immunofluorescence staining was quantified using ImageJ software. Briefly, the same threshold was set for all images, and the positive staining area was divided by the total field area. The relative density was expressed as arbitrary units (AU). * *p* < 0.05, ** *p* < 0.01, *** *p* < 0.001; *n* = 6. Scale bar: 100 μm.

Hematoxylin and eosin (H&E) staining showed that the jejunal villi in the CON and CON_PLE groups were structurally intact and regularly arranged, with compact and orderly epithelial cells (Figure 2a). The jejunal villi in the HFD group were significantly shortened, sparse and disorganized, suggesting that a high-fat diet could induce structural damage to the intestinal mucosa. Compared with the HFD group, HFD_PLE, HFD_SE and HFD_OCA all improved the morphology of jejunal villi to varying degrees, among which the reparative effect of HFD_PLE was the most obvious. Quantitative results indicated that supplementation with pepper leaf extracts (PLE) significantly increased jejunal villus length. Compared with the HFD group, villus length was extremely significantly increased in the HFD_PLE group (*p* < 0.001) and significantly increased in the HFD_OCA group (*p* < 0.05) (Figure 2e).

Periodic acid-Schiff (PAS) staining showed that the CON and CON_PLE groups had abundant goblet cells and sufficient mucus secretion (Figure 2b). The number of goblet cells in the HFD group was significantly decreased, accompanied by a weakened mucus barrier. Compared with the HFD group, the number of goblet cells was extremely significantly increased in the HFD_PLE group (*p* < 0.01), while HFD_SE and HFD_OCA showed an upward trend without statistical significance (*p* > 0.05) (Figure 2f). Quantitative results of crypt depth demonstrated that PLE supplementation effectively elevated crypt depth. Compared with the HFD group, crypt depth was extremely significantly increased in the HFD_PLE group (*p* < 0.01), while HFD_SE and HFD_OCA exhibited a slight recovery but did not reach a significant level (*p* > 0.05) (Figure 2g).

Immunofluorescence staining for Occludin revealed that the CON and CON_PLE groups displayed continuous and bright fluorescent signals (Figure 2c). The fluorescence intensity was weakened in the HFD group, indicating impaired intestinal tight junctions. Compared with the HFD group, the fluorescence intensity of Occludin in the HFD_PLE group showed an upward trend without statistical significance (*p* > 0.05), while that in the HFD_OCA group was slightly decreased (Figure 2d).

**Figure 3:**
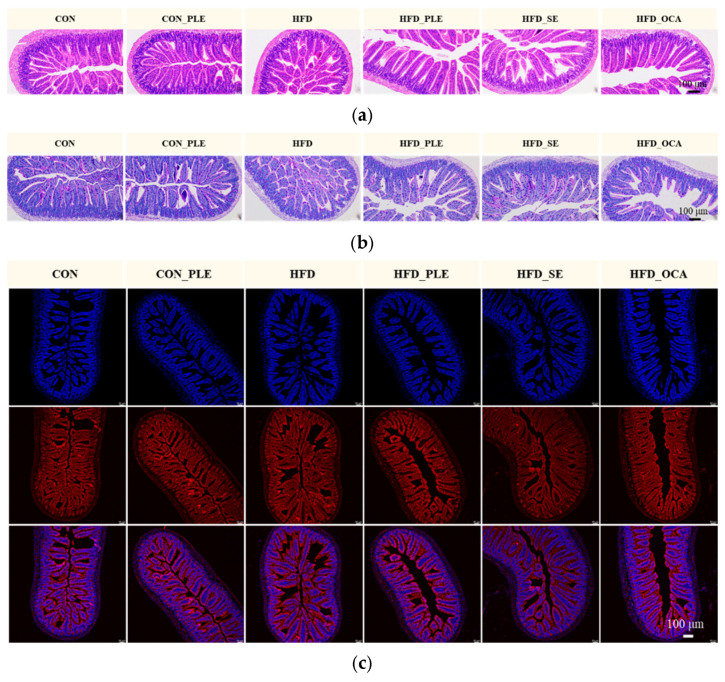

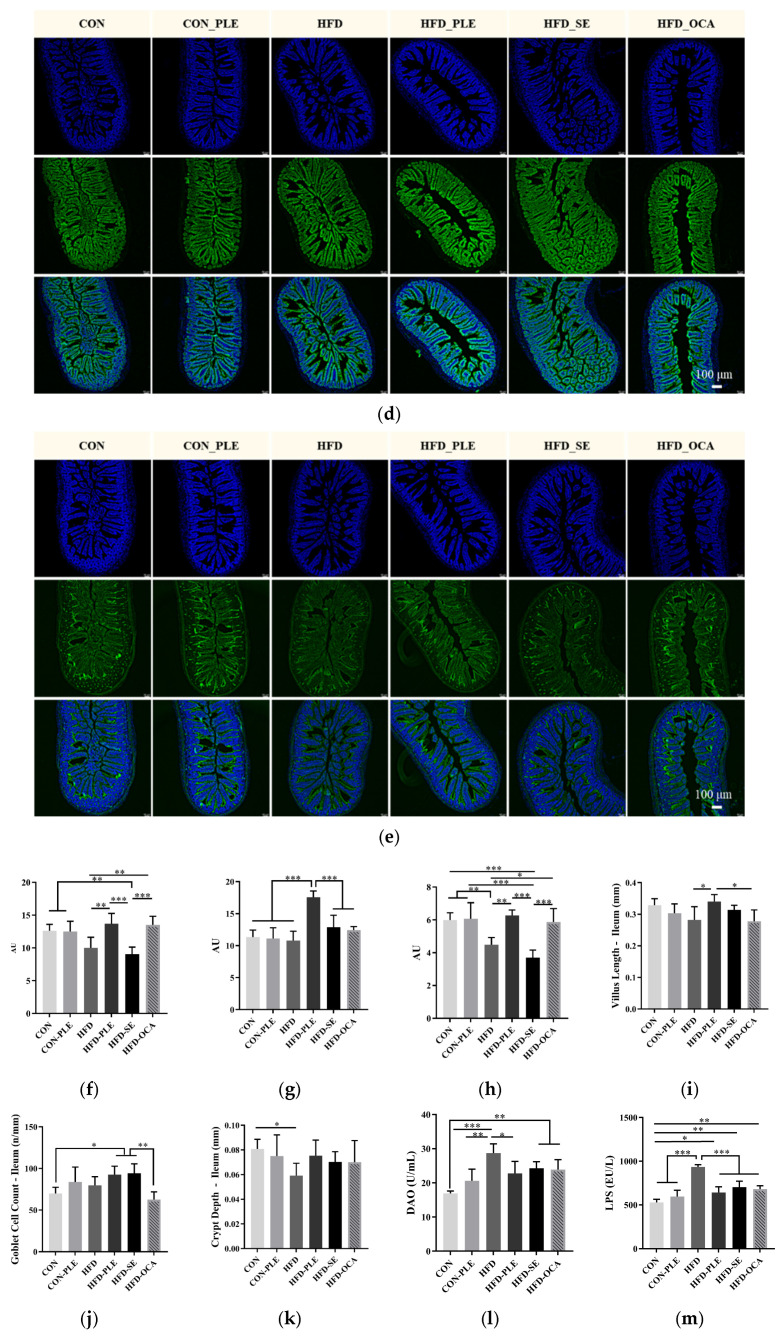
Pepper leaf extracts (PLE) improved intestinal barrier in ileum. (**a**) Representative graph of ileum H&E staining (100×). (**b**) Representative graph of ileum PAS staining (100×). (**c**) Representative graph of ileum Occludin immunofluorescence (Blue: DAPI (nucleus); red: Occludin; magenta: merged signals; 100×). (**d**) Representative graph of ileum Claudin immunofluorescence (Blue: DAPI (nucleus); green: Claudin; magenta: merged signals; 100×). (**e**) Representative graph of ileum ZO-1 immunofluorescence (Blue: DAPI (nucleus); green: ZO-1; magenta: merged signals; 100×). (**f**) Immunofluorescence positive surface density of Occludin protein in ileum. (**g**) Immunofluorescence positive surface density of Claudin protein in ileum. (**h**) Immunofluorescence positive surface density of ZO-1 protein in ileum. (**i**) Villus length. (**j**) Goblet cell count. (**k**) Crypt depth. (**l**) Serum diamine oxidase (DAO) content. (**m**) Serum lipopolysaccharide (LPS) content. The positive surface density of immunofluorescence staining was quantified using ImageJ software. Briefly, the same threshold was set for all images, and the positive staining area was divided by the total field area. The relative density was expressed as arbitrary units (AU). * *p* < 0.05, ** *p* < 0.01, *** *p* < 0.001; *n* = 6. Scale bar: 100 μm.

Hematoxylin and eosin (H&E) staining revealed that the ileal villi in the CON and CON_PLE groups were neatly arranged and structurally intact, with regularly shaped epithelial cells (Figure 3a). The ileal villi in the HFD group exhibited obvious atrophy and disorganized arrangement, indicating that high-fat diet induced structural damage to the ileal mucosa. Compared with the HFD group, HFD_PLE, HFD_SE and HFD_OCA all improved villus morphology to varying degrees, among which the protective effect of HFD_PLE was the most prominent. Quantitative analysis demonstrated that, compared with the HFD group, ileal villus length was significantly increased in the HFD_PLE group (*p* < 0.05), while HFD_SE showed an upward trend without statistical significance (*p* > 0.05) (Figure 3i).

Periodic acid-Schiff (PAS) staining showed that the CON and CON_PLE groups possessed sufficient goblet cells and abundant mucus secretion (Figure 3b). The number of goblet cells was decreased and the function of the mucus barrier was impaired in the HFD group. Compared with the HFD group, the number of goblet cells showed an upward trend in the HFD_PLE and HFD_SE groups and a downward trend in the HFD_OCA group, with no statistically significant differences among all groups (*p* > 0.05) (Figure 3j). Quantitative results of crypt depth indicated that all intervention groups showed an upward trend compared with the HFD group, but the differences did not reach statistical significance (Figure 3k).

Occludin, Claudin and ZO-1 are the core proteins constituting intestinal tight junctions. Immunofluorescence results showed that the three proteins exhibited strong and continuously distributed fluorescent signals in the CON and CON_PLE groups; the fluorescence intensity was decreased in the HFD group, suggesting the collapse of tight junction structures and a significant increase in intestinal permeability (Figure 3c–e).

Compared with the HFD group, the fluorescence intensity of ileal Occludin was extremely significantly increased in the HFD_PLE and HFD_OCA groups (*p* < 0.01), while no significant change was observed in the HFD_SE group (*p* > 0.05) (Figure 3f). Compared with the HFD group, the fluorescence intensity of ileal Claudin was extremely significantly increased in the HFD_PLE group (*p* < 0.001) (Figure 3g). For the fluorescence intensity of ZO-1, an extremely significant increase was observed in the HFD_PLE group (*p* < 0.01) and a significant increase in the HFD_OCA group (*p* < 0.05) (Figure 3h).

Compared with the CON group, serum DAO and LPS levels were extremely significantly increased in HFD mice (*p* < 0.001). Compared with the HFD group, serum DAO level was significantly decreased in the HFD_PLE group (*p* < 0.05), while HFD_SE and HFD_OCA showed a downward trend without statistical significance (*p* > 0.05); serum LPS levels were extremely significantly decreased in the HFD_PLE, HFD_SE and HFD_OCA groups, among which HFD_PLE exerted the optimal effect (Figure 3l–m).

### 3.3. Pepper Leaf Extracts Modulate Gut Microbiota

**Figure 4:**
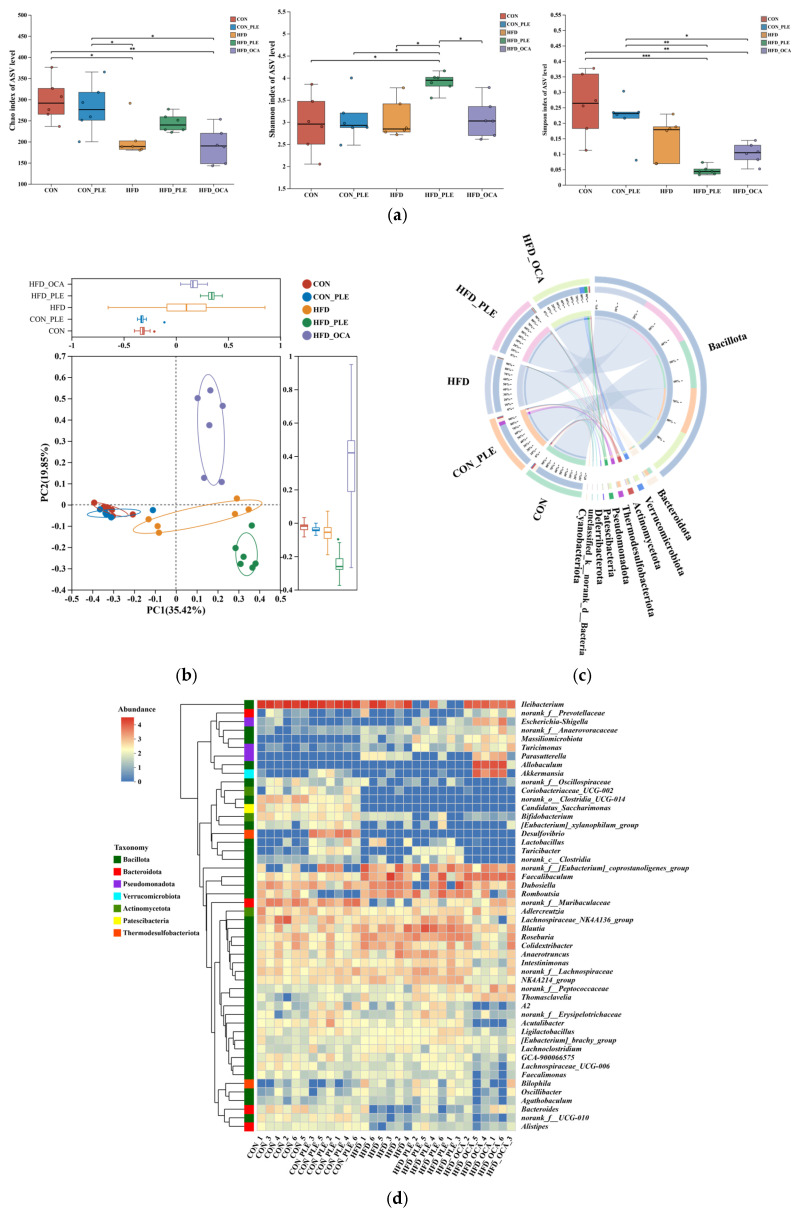

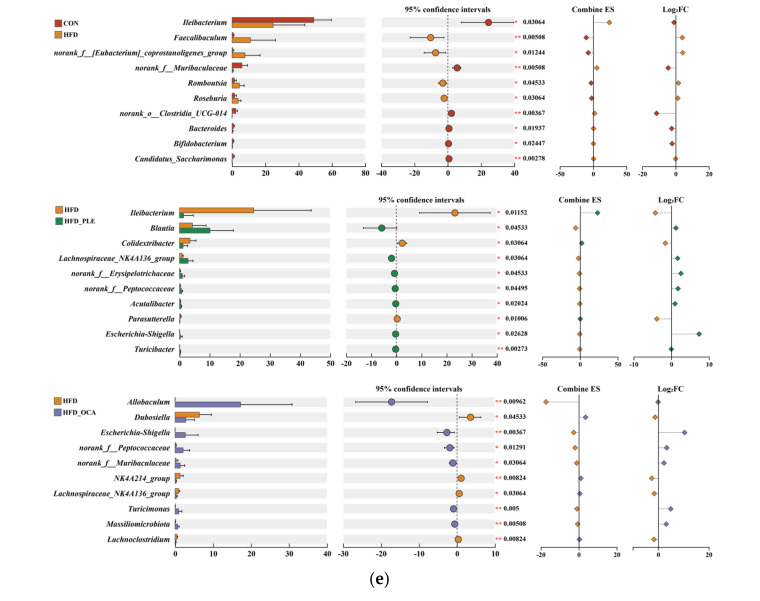

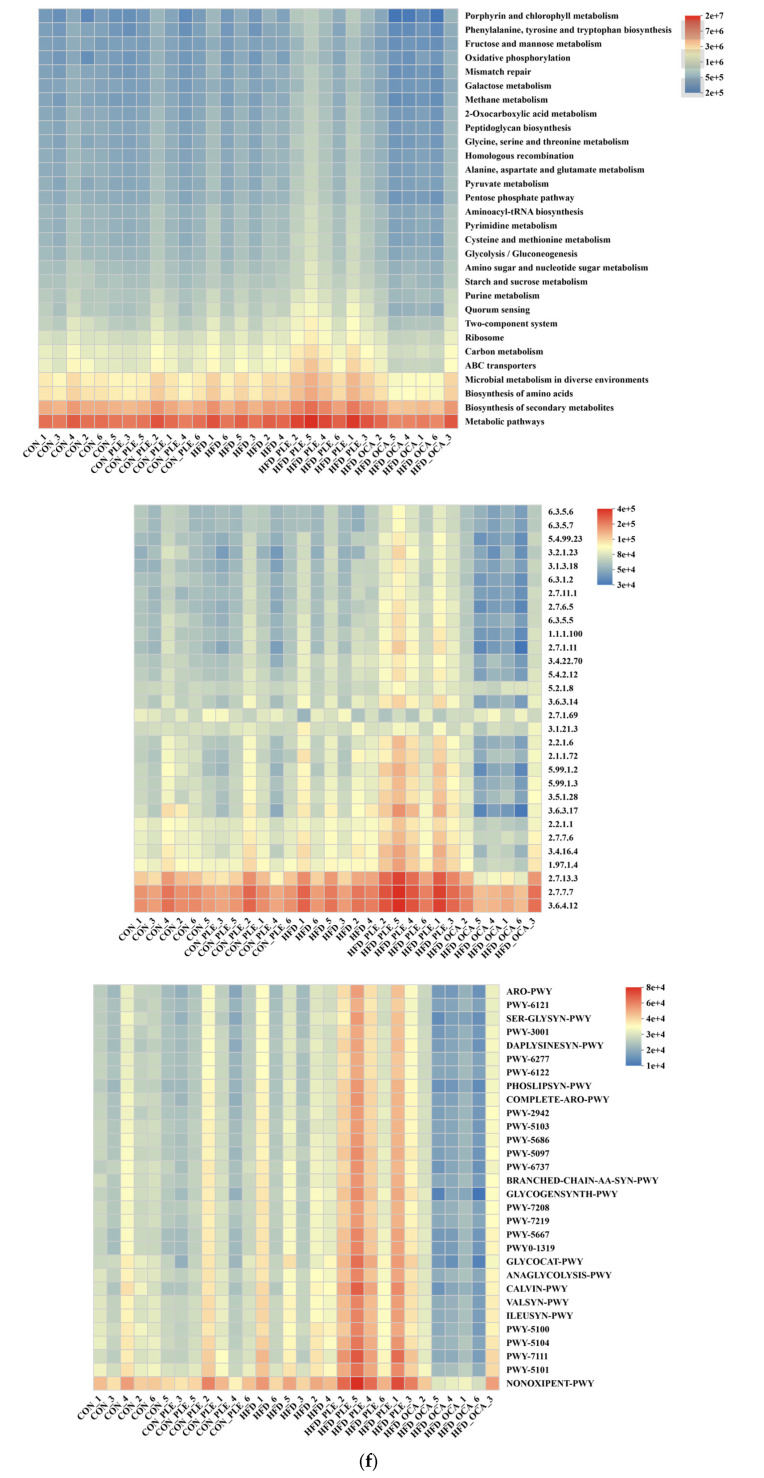
Pepper leaf extracts (PLE) improved gut microbiota. (**a**) α-diversity analysis. (**b**) β-diversity analysis (PCoA). (**c**) Microbial community composition-phylum level. (**d**) Clustered heatmap-genus level. (**e**) Differential analysis between two groups-genus level. (**f**) PICRUSt2 functional prediction (from top to bottom: KEGG-Level 3, Enzyme, MetaCyc-pathway). * *p* < 0.05, ** *p* < 0.01, *** *p* < 0.001; *n* = 6.

Compared with the CON group, the HFD group exhibited a significantly reduced Chao index (*p* < 0.05), whereas the Shannon and Simpson indices showed decreasing trends without reaching statistical significance (*p* > 0.05). In contrast, the HFD_PLE group displayed a non-significant upward trend in the Chao index (*p* > 0.05) and a markedly elevated Shannon index (*p* < 0.05) relative to the HFD group, with no significant alteration in the Simpson index (*p* > 0.05) (Figure 4a). Principal coordinates analysis (PCoA) based on Bray–Curtis dissimilarity distances further demonstrated a distinct separation in the global gut microbial community structure between the CON and HFD groups (Figure 4b).

At the phylum level (Figure 4c), compared with the CON group, the HFD group showed a marked increase in Bacillota and a significant reduction in Bacteroidota and Actinomycetota. PLE supplementation reversed this phylum-level imbalance, reducing Bacillota while increasing Bacteroidota and Actinomycetota abundances.

At the genus level (Figure 4d,e), relative to the CON group, the HFD group displayed notably enriched relative abundances of *Faecalibaculum*, *norank_f_[Eubacterium]_coprostanoligenes_group*, *Romboutsia* and *Roseburia*, alongside significantly diminished relative abundances of *Ileibacterium*, *norank_f_Muribaculaceae*, *norank_o_Clostridia_UCG-014*, *Bacteroides*, *Bifidobacterium* and *Candidatus_Saccharimonas*. In comparison with the HFD group, supplementation with PLE resulted in elevated relative abundances of *Blautia*, *Lachnospiraceae_NK4A136_group*, *norank_f_Erysipelotrichaceae*, *norank_f_Peptococcaceae*, *Acutalibacter*, *Escherichia-Shigella* and *Turicibacter*, as well as reduced relative abundances of *Ileibacterium*, *Parasutterella* and *Colidextribacter*. Meanwhile, treatment with OCA increased the relative abundances of *Allobaculum*, *Escherichia-Shigella*, *norank_f_Peptococcaceae*, *norank_f_Muribaculaceae*, *Turicimonas* and *Massiliomicrobiota*, while decreasing those of *Dubosiella*, *Lachnospiraceae_NK4A136_group*, *NK4A214_group* and *Lachnoclostridium*.

PICRUSt2 (version 2.2.0) functional prediction (Figure 4f) revealed that PLE significantly enriched Carbon metabolism, Biosynthesis of amino acids, Purine metabolism, ABC transporters, quorum sensing and two-component systems relative to the HFD group. PLE selectively enriched key enzymes (EC 1.1.1.100, EC 2.2.1.1, EC 2.7.13.3) and cognate MetaCyc pathways (ARO-PWY, NONOXIPENT-PWY, PWY-7219, COMPLETE-ARO-PWY, ANAGLYCOLYSIS-PWY).

### 3.4. Pepper Leaf Extracts Modulate Cecal Bile Acid Profiles

To investigate the regulatory effects of cecal gut microbiota on secondary bile acid metabolism, bile acid profiles in cecal contents were quantitatively determined.

As shown in Table 2, markedly elevated levels of primary free bile acids, primary conjugated bile acids, secondary free bile acids, secondary conjugated bile acids, total primary bile acids, total secondary bile acids, and total bile acids were observed in the HFD group relative to the CON group (all *p* < 0.05). Additionally, the primary/secondary bile acid ratio and conjugated/free bile acid ratio were both significantly increased in the HFD group (*p* < 0.05). These results suggest that long-term high-fat diet intake severely disrupts cecal bile acid metabolism, characterized by excessive accumulation of both primary and secondary bile acids, which may be closely associated with HFD-induced gut microbiota dysbiosis and impaired bile acid transformation and excretion.

Pepper leaf extracts (PLE) intervention significantly reversed HFD-induced bile acid abnormalities: primary free bile acids, primary conjugated bile acids, secondary free bile acids, secondary conjugated bile acids, total primary bile acids, total secondary bile acids, and total bile acids were all significantly decreased in the HFD_PLE group (all *p* < 0.05) relative to the HFD group. The primary/secondary bile acid ratio was also reduced to a level close to that of the CON group, whereas the conjugated/free bile acid ratio was significantly increased (*p* < 0.05). This indicates that PLE effectively restores bile acid homeostasis by reducing the overall accumulation of bile acids and normalizing the balance between primary and secondary bile acids, which may be mediated by the regulatory effect of PLE on cecal gut microbiota and their bile acid-metabolizing functions.

Relative to the HFD group, the HFD_OCA group showed a remarkable reduction in primary free bile acids, primary conjugated bile acids, secondary free bile acids, total primary bile acids, total secondary bile acids, and total bile acids (all *p* < 0.05), along with a significant decrease in the primary/secondary bile acid ratio (*p* < 0.05). Conversely, secondary conjugated bile acid levels and the conjugated/free bile acid ratio were significantly elevated in the HFD_OCA group (*p* < 0.05), indicating a specific regulatory effect of OCA on cecal bile acid profiles to alleviate HFD-induced metabolic disruption.

### 3.5. Pepper Leaf Extracts Regulate Fat Metabolism

**Figure 5:**
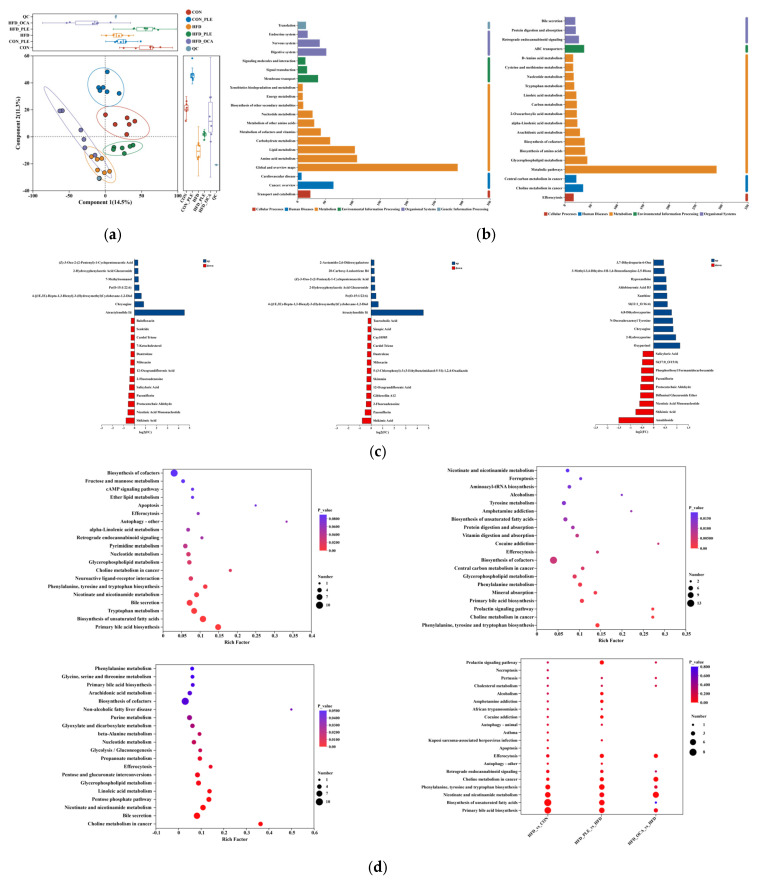
Pepper leaf extracts (PLE) improved metabolism. (**a**) PLS-DA. (**b**) KEGG pathway enrichment of all samples (from left to right: Level 2, Level 3; Functional classification of genes. Colors represent different KEGG categories: red (Cellular Processes), blue (Human Diseases), orange (Metabolism), green (Environmental Information Processing), purple (Organismal Systems), and light blue (Genetic Information Processing)). (**c**) Differential metabolites (CON vs. HFD, HFD vs. HFD_PLE, HFD vs. HFD_OCA). (**d**) KEGG pathway enrichment (from left to right: CON vs. HFD, HFD vs. HFD_PLE, HFD vs. HFD_OCA, multiple group comparison. *n* = 6.

Partial Least Squares-Discriminant Analysis (PLS-DA) score plots (Figure 5a, mixed dataset) exhibited a distinct separation between the CON and HFD groups. Relative to the HFD group, the HFD_OCA group displayed negligible separation, while the HFD_PLE group showed a marked divergence and clustered closely with the CON group. KEGG Level 2 and 3 pathway enrichment analysis showed that perturbed metabolites were predominantly enriched in carbohydrate metabolism, lipid metabolism, amino acid metabolism, nucleotide metabolism, energy metabolism, “Xenobiotics biodegradation and metabolism”, “Bile secretion” and “ABC transporters” (Figure 5b).

Specifically, relative to the CON group, the HFD group had significantly increased levels of Z-3-oxo-2-(2-pentenyl)-1-cyclopenteneacetic acid, 2-hydroxyphenylacetic acid glucuronide, 7-methylrosmanol, PE(O-15:1/22:6), 4-[(1E,3E)-hepta-1,3-dienyl]-3-(hydroxymethyl)cyclohexane-1,2-diol, chrysogine, and atractylenilide III (all *p* < 0.05). In contrast, the levels of senktide, cardol triene, 7-ketocholesterol, 12-oxograndiflorenic acid, 2-fluoroadenosine, salicyluric acid, paeoniflorin, protocatechuic aldehyde, nicotinic acid mononucleotide, and shikimic acid were significantly decreased in the HFD group (all *p* < 0.05).

Relative to the HFD group, the HFD_PLE group had significantly increased levels of 2-acetamido-2,6-dideoxygalactose, 20-carboxy-leukotriene B4, (Z)-3-oxo-2-(2-pentenyl)-1-cyclopenteneacetic acid, 2-hydroxyphenylacetic acid glucuronide, PE(O-15:1/22:6), 4-[(1E,3E)-hepta-1,3-dienyl]-3-(hydroxymethyl)cyclohexane-1,2-diol, and atractylenilide III (all *p* < 0.05). Meanwhile, the levels of taurocholic acid, sinapic acid, cardol triene, skimmin, 12-oxograndiflorenic acid, gibberellin A12, 2-fluoroadenosine, paeoniflorin, and shikimic acid were significantly decreased in the HFD_PLE group (all *p* < 0.05).

Compared with the HFD group, the HFD_COA group showed significantly increased levels of 3,7-dihydropurin-6-one, hypoxanthine, aldobiouronic acid D3, xanthine, SI(12:1-O/16:4), 6,8-dihydroxypurine, N-docosahexaenoyl tyrosine, chrysogine, 2-hydroxypurine, and oxypurinol (all *p* < 0.05).On the contrary, the levels of salicyluric acid, SI(17:0-O/15:0), phosphoribosyl formamidocarboxamide, paeoniflorin, protocatechuic aldehyde, nicotinic acid mononucleotide, shikimic acid, and amabiloside were significantly decreased in the HFD_COA group (all *p* < 0.05) (Figure 5c).

KEGG pathway enrichment analysis showed that in the HFD vs. CON comparison, the differential metabolites were significantly enriched in pathways intimately associated with lipid metabolism (primary bile acid biosynthesis, bile secretion, glycerophospholipid metabolism, ether lipid metabolism, biosynthesis of unsaturated fatty acids, and alpha-linolenic acid metabolism), amino acid metabolism (phenylalanine/tyrosine/tryptophan biosynthesis and tryptophan metabolism), carbohydrate metabolism (fructose and mannose metabolism), nucleotide metabolism (nucleotide metabolism and pyrimidine metabolism), and nicotinate and nicotinamide metabolism.

Following PLE intervention (HFD_PLE vs. HFD), the regulated metabolites were mainly enriched in pathways involved in amino acid metabolism (phenylalanine/tyrosine/tryptophan biosynthesis, phenylalanine metabolism, and tyrosine metabolism), lipid metabolism (primary bile acid biosynthesis, glycerophospholipid metabolism, and biosynthesis of unsaturated fatty acids), and nicotinate and nicotinamide metabolism. In addition, aminoacyl-tRNA biosynthesis, vitamin digestion and absorption, and protein digestion and absorption were also significantly enriched.

In the HFD_OCA vs. HFD comparison, OCA-modulated metabolites were enriched in pathways related to lipid metabolism (primary bile acid biosynthesis, bile secretion, glycerophospholipid metabolism, linoleic acid metabolism, and arachidonic acid metabolism), energy and carbohydrate metabolism (pentose phosphate pathway, pentose and glucuronate interconversions, glycolysis/gluconeogenesis, and propanoate metabolism), nucleotide metabolism (nucleotide metabolism and purine metabolism), amino acid metabolism (glycine, serine and threonine metabolism, and phenylalanine metabolism), nicotinate and nicotinamide metabolism, and non-alcoholic fatty liver disease (Figure 5d).

## 4. Discussion

HFD feeding induced overt obesity in mice, characterized by increased body weight, excessive body weight gain, and elevated food efficiency ratio (FER), a key indicator that reflected the efficiency of dietary energy conversion into body mass [19]. All three interventions alleviated obese phenotypes, though their underlying mechanisms differed substantially. PLE exhibited the most potent anti-obesity effect, with a greater reduction in body weight compared with OCA and SE, alongside a marked decrease in FER. Importantly, PLE and SE induced weight loss was independent of appetite suppression, as no differences in energy intake were detected among the HFD_PLE, HFD_SE, and HFD groups. In contrast, OCA mediated weight loss was partially attributed to appetite suppression, as reflected by reduced energy intake relative to the HFD group, which may limit its long-term application due to potential nutritional deficiencies. The distinct mechanisms of weight loss highlight the potential advantage of PLE for long-term obesity management, as its effect appears independent of appetite suppression.

The anti-obesity effect of PLE was further supported by remodeling of adipose tissue, a key contributor to HFD-induced metabolic dysfunction. HFD feeding induced severe whitening of brown adipose tissue (sBAT) [20], characterized by loss of typical multilocular lipid droplet structure, vacuolar degeneration, and impaired thermogenic capacity, factors that collectively contribute to energy surplus and obesity development in this murine model. Meanwhile, HFD exposure caused significant hypertrophy of white adipose tissue (WAT) at three critical sites (epididymal, inguinal, and perirenal), which was closely associated with promotion of adipose inflammation, lipotoxicity, and systemic insulin resistance [21]. PLE treatment decreased BAT relative weight and markedly reduced the relative weight of all three WAT depots, indicating restoration of BAT thermogenic function and inhibition of WAT expansion. This observation further elucidated the reduced FER in the PLE group: enhanced BAT mediated non-shivering thermogenesis promoted energy expenditure rather than adipose storage [22]. OCA exerted similar but less potent effects on adipose tissue remodeling, while SE only reduced epididymal WAT weight without meaningful impact on body weight gain. These differences highlighted that SE exhibited relatively weaker efficacy compared with PLE, which demonstrated a broader regulatory effect on adipose tissue homeostasis. This discrepancy may be attributed to differences in their respective phytochemical compositions or bioactive compound concentrations.

Insulin resistance was one of the pathophysiological bases of HFD-induced metabolic disorders, and HFD feeding impaired glucose tolerance, reduced insulin sensitivity, and increased the HOMA-IR index. All three interventions decreased the HOMA-IR index, but PLE exhibited the most potent effect on glucose homeostasis and insulin sensitivity.

The robust improvement of insulin resistance by PLE was a synergistic effect of multi-organ regulatory actions of this extract: (1) PLE reduced WAT hypertrophy and adipose inflammation, thereby alleviating peripheral insulin resistance; (2) it ameliorated hepatic steatosis and reduced hepatic gluconeogenesis, improving hepatic insulin sensitivity; (3) it restored intestinal barrier function, reducing LPS-induced systemic inflammation; (4) it regulated the gut microbiota and bile acid metabolism. By ameliorating pathology in multiple organs ncluding adipose tissue, liver, and intestine PLE addressed the complex, systemic nature of insulin resistance, demonstrating a key advantage over single-target interventions such as OCA.

Hepatic steatosis and its progression were closely linked to systemic metabolic dysregulation induced by HFD [23]. HFD feeding resulted in marked hepatic lipid accumulation in mice, as evidenced by hepatomegaly, pale hepatic coloration, disrupted hepatic lobule architecture, extensive hepatocellular steatosis, hepatocyte ballooning degeneration, focal inflammatory cell infiltration, and early-stage fibrotic lesions [24]. Quantitative assessment further verified that liver weight, as well as hepatic total cholesterol (TC) and triglyceride (TG) levels, were elevated in the HFD group compared with the CON group. All three interventions alleviated hepatic steatosis, with PLE demonstrating the most prominent effect, as reflected by reduced lipid droplet accumulation, improved hepatic lobule structure, and minimal inflammatory and fibrotic lesions. The pronounced hepatoprotective effect of PLE was closely associated with its regulatory effects on intestinal barrier function and the gut microbiota-bile acid axis.

Intestinal barrier dysfunction was a key mediator of HFD induced hepatic steatosis and inflammation in this murine model, serving as a critical link between gut dysbiosis and hepatic metabolic damage [25,26]. HFD feeding impaired the structural integrity of jejunal and ileal mucosa, characterized by villus shortening and disorganization, reduced goblet cell numbers (indicating impaired mucus barrier function), and downregulated expression of tight junction proteins (Occludin, Claudin, ZO-1), ultimately leading to increased intestinal permeability [27,28,29]. This phenomenon was directly confirmed by elevated serum diamine oxidase (DAO) and lipopolysaccharide (LPS) levels in the HFD group: DAO served as a specific marker of intestinal epithelial cell damage in this context, while LPS was a gut-derived endotoxin that entered the systemic circulation through a compromised intestinal barrier, thereby triggering hepatic inflammation and lipotoxicity [30,31,32]. PLE intervention exhibited the most potent restorative effect on intestinal barrier function, as it increased jejunal and ileal villus length, elevated ileal goblet cell numbers and crypt depth, and upregulated the expression of ileal tight junction proteins. Furthermore, PLE reduced serum DAO and LPS levels, confirming its ability to improve intestinal barrier integrity and reduce gut leakage. OCA also improved ileal tight junction protein expression and reduced serum LPS levels, but exerted no meaningful effects on DAO levels or goblet cell numbers, while SE showed only mild protective effects on intestinal morphology. These observations confirmed that the superior hepatoprotective effect of PLE was partially attributed to its robust restoration of intestinal barrier function, which mitigated LPS-induced hepatic inflammation and lipotoxicity, an effect that distinguished PLE from SE and OCA.

The gut microbiota serves as a key regulator of intestinal barrier function and bile acid metabolism, and HFD feeding significantly disrupted the gut microbial community structure in the present study [33]. HFD exposure reduced gut microbial richness, and induced phylum-level dysbiosis (increased abundance of Bacillota, decreased abundances of Bacteroidota and Actinomycetota) as well as genus-level perturbations (enriched *Faecalibaculum* and *Romboutsia*; depleted *Bacteroides* and *Bifidobacterium*) [34,35]. These changes indicated that HFD-induced gut dysbiosis contributed to intestinal barrier damage and metabolic disorders [36]. PLE intervention effectively reversed this dysbiosis, increased microbial diversity, reduced Bacillota abundance, and elevated Bacteroidota and Actinomycetota levels. At the genus level, PLE enriched beneficial taxa associated with short-chain fatty acid (SCFA) production and intestinal barrier protection, including *Blautia* and *Lachnospiraceae_NK4A136_group*, while reducing the abundance of potentially pathogenic taxa, including *Ileibacterium* and *Parasutterella* [37,38]. OCA also remodeled the gut microbiota in a distinct pattern, with enriched *Allobaculum* and depleted *Dubosiella*. This observation revealed divergent microbial regulatory mechanisms between PLE and OCA. These distinct microbial remodeling patterns may underlie the differences in therapeutic efficacy between the two interventions.

The regulatory role of the gut microbiota in metabolic homeostasis was directly confirmed by changes in cecal bile acid profiles, as bile acids act as key signaling molecules linking the gut microbiota to host metabolism, with gut bacteria mediating the conversion of primary bile acids to secondary bile acids [39]. HFD feeding severely disrupted bile acid metabolism, increasing levels of primary free, primary conjugated, secondary free, secondary conjugated, and total bile acids, and elevating the primary/secondary bile acid ratio and conjugated/free bile acid ratio. This abnormal accumulation of bile acids was closely associated with HFD-induced gut dysbiosis, as depletion of Bacteroidota and Actinomycetota, key taxa involved in bile acid transformation, impaired bile acid excretion and metabolism [34,37,40]. PLE intervention significantly reversed these abnormalities, reducing all classes of bile acids to levels comparable to the CON group, and normalizing the primary/secondary bile acid ratio, confirming the ability of this extract to restore bile acid homeostasis. OCA also reduced total bile acid levels, but increased secondary conjugated bile acid levels and the conjugated/free bile acid ratio, reflecting direct regulation of hepatic bile acid synthesis by OCA via farnesoid X receptor (FXR) mediation [41], which was distinct from the microbiota-dependent regulatory mechanism of PLE. These results demonstrated that PLE-induced gut microbiota remodeling was a key mechanism underlying regulation of bile acid metabolism by this extract, which in turn contributed to alleviation of hepatic steatosis and metabolic disorders. The microbiota-bile acid axis thus represented a central regulatory pathway through which PLE exerted therapeutic effects.

PICRUSt2 functional prediction further supported the role of the gut microbiota in the therapeutic effect of PLE: PLE enriched microbial pathways related to carbon metabolism, amino acid biosynthesis, and ABC transporters, functions closely associated with nutrient metabolism and bile acid transport [42,43,44]. This observation was consistent with metabolomic findings, forming a gut microbiota-bile acid-metabolome regulatory axis that integrated microbial function, bile acid signaling, and systemic metabolism.

Partial Least Squares-Discriminant Analysis (PLS-DA) showed a clear separation between the CON and HFD groups, while PLE intervention restored the metabolic profile to a trend similar to that of the CON group, with OCA exerting a weaker effect. This observation indicated that PLE was more effective than OCA in reversing HFD-induced metabolic perturbations.

2-Hydroxyphenylacetic acid glucuronide is a marker of dual pathological damage induced by HFD: intestinal barrier dysfunction and oxidative stress. PE(O-15:1/22:6) indicates impaired cell membrane structure [45], which further exacerbates apoptosis and amplifies inflammatory responses. Increased polygonatum levels indicate gut microbiota dysbiosis [46], which may exacerbate intestinal inflammation and metabolic disorders. Downregulated beneficial metabolites (Cardol triene, salicylic acid, paeoniflorin, protocatechualdehyde) impair host anti-inflammatory and antioxidant capacities and perpetuate the inflammation-oxidative stress vicious cycle [47,48,49,50,51]. Depletion of nicotinamide mononucleotide leads to NAD+ pool exhaustion, resulting in dual adverse effects: impaired energy metabolism efficiency and weakened SIRT1-mediated antioxidant and anti-inflammatory signaling [52]. In summary, HFD induced multidimensional metabolic network dysregulation, characterized by accumulation of pro-inflammatory metabolites, reduction in anti-inflammatory/antioxidant metabolites, abnormal bile acid metabolism, and intestinal barrier damage. Consistent with its regulatory effects on lipid and bile acid metabolism, PLE increased the level of glycerophospholipid PE(O-15:1/22:6) involved in lipid homeostasis and decreased the level of taurocholic acid (a primary bile acid). Increased abundance of 2-acetamido-2,6-dideoxygalactose indicates intestinal barrier restoration through mucin biosynthesis [53]. In contrast, OCA regulated metabolites involved in fatty acid metabolism, reflecting the unique therapeutic targets of this agonist. Differential metabolite analysis indicated that both OCA and PLE effectively improved HFD-induced metabolic disorders, with the consistency between metabolomics and phenotypic improvements (obesity, hepatic steatosis, insulin resistance) confirming their multi-pathway regulatory effects.

KEGG pathway enrichment analysis further elucidated the metabolic mechanisms underlying the therapeutic effects of the interventions. In the HFD vs. CON comparison, differential metabolites were enriched in lipid metabolism (primary bile acid biosynthesis, glycerophospholipid metabolism), amino acid metabolism (phenylalanine/tyrosine/tryptophan biosynthesis), carbohydrate metabolism (fructose and mannose metabolism), and nucleotide metabolism, which was consistent with HFD-induced metabolic perturbations [54,55,56]. KEGG pathway enrichment analysis indicated that PLE intervention primarily modulated pathways related to bile acid, amino acid, and energy metabolism, as well as nutrient digestion and absorption. This multi-pathway regulation is consistent with its observed effects on the gut microbiota, bile acid metabolism, and intestinal barrier function. This further suggests that the therapeutic effect of PLE may be mediated, at least in part, by metabolic reprogramming driven by alterations in gut microbial function. Notably, the enrichment of the “xenobiotics biodegradation and metabolism” pathway raises the possibility that components of PLE, such as chlorophyll, may be metabolized by the gut microbiota into bioactive intermediates. Indeed, previous studies have shown that chlorophyll derivatives like pheophytin can inhibit fatty acid synthase (FAS) activity [57,58], suggesting a potential mechanism that warrants further investigation. OCA-modulated metabolites were enriched in lipid metabolism, glucose metabolism (glycolysis/gluconeogenesis), and NAFLD-related pathways, which is also consistent with its role as a farnesoid X receptor (FXR) agonist targeting hepatic lipid metabolism.

## 5. Conclusions

In this study, we systematically investigated the therapeutic effects and underlying mechanisms of pepper leaf extracts (PLEs) on HFD-induced metabolic dysfunction in mice through phenotypic, histological, microbial, bile acid profile, and metabolomic analyses. PLE effectively ameliorated HFD-induced obesity, adipose tissue remodeling disorders, hepatic steatosis, insulin resistance, and intestinal barrier damage in mice, exhibiting superior therapeutic efficacy compared with spinach extracts (SEs) and the positive control obeticholic acid (OCA). The therapeutic mechanisms of PLE were associated with its regulation of the gut microbiota–bile acid–metabolome axis: PLE remodeled the gut microbiota, restored intestinal barrier integrity, normalized bile acid metabolism, and modulated multiple metabolic pathways, thereby improving systemic metabolic homeostasis. These findings provide a foundation for PLE as a novel, safe, and effective functional base material for the prevention and alleviation of HFD-induced metabolic disorders, offering a new strategy for the dietary management of obesity and its associated metabolic complications.

## Figures and Tables

**Table 1:** Experiment design and treatments.

Group	Abbreviation	Diet	Gavage Substance
Control	CON	XT04	0.5% CMC-Na aqueous solution
Control + pepper leaf extracts	CON_PLE	XT304 + 2% pepper leaf extracts	0.5% CMC-Na aqueous solution
High-fat diet	HFD	XT310	0.5% CMC-Na aqueous solution
High-fat diet + pepper leaf extracts	HFD_PLE	XT310 + 2% pepper leaf extracts	0.5% CMC-Na aqueous solution
High-fat diet + spinach extracts	HFD_SE	XT310 + 1.98% spinach extracts	0.5% CMC-Na aqueous solution
High-fat diet + obeticholic acid	HFD_OCA	XT310 + obeticholic acid	Obeticholic acid ^1^ dissolved in 0.5% CMC-Na aqueous solution

^1^ The gavage dosage of obeticholic acid was 10 mg/kg body weight per day.

**Table 2:** Contents of bile acids in cecal contents.

Category	Name	Content (ng/mg)				
		CON	CON_PLE	HFD	HFD_PLE	HFD_OCA
Primary free bile acids	Cholic acid	6.159 ± 0.315 ^c^	7.405 ± 0.433 ^c^	58.648 ± 2.673 ^b^	26.532 ± 4.031 ^c^	113.927 ± 19.496 ^a^
	Chenodeoxycholic acid	6.517 ± 1.132 ^d^	6.190 ± 0.238 ^d^	80.124 ± 5.531 ^a^	26.974 ± 2.144 ^c^	45.975 ± 9.939 ^b^
	Allocholic Acid	2.357 ± 0.171 ^d^	1.553 ± 0.218 ^d^	99.568 ± 4.901 ^a^	34.075 ± 5.502 ^c^	65.913 ± 0.647 ^b^
	Apocholic acid	0.101 ± 0.010 ^b^	0.197 ± 0.064 ^b^	1.165 ± 0.382 ^a^	0.596 ± 0.164 ^b^	0.345 ± 0.033 ^b^
	Hyocholic acid	0.980 ± 0.157 ^cd^	0.920 ± 0.020 ^d^	5.584 ± 0.577 ^a^	1.738 ± 0.222 ^c^	2.753 ± 0.152 ^b^
	Hyodeoxycholic acid	24.124 ± 1.187 ^b^	24.021 ± 3.287 ^b^	71.354 ± 1.525 ^a^	14.909 ± 1.767 ^c^	8.906 ± 0.308 ^d^
	Norcholic acid	0.311 ± 0.086 ^c^	0.382 ± 0.086 ^c^	1.476 ± 0.425 ^a^	1.178 ± 0.203 ^ab^	0.646 ± 0.217 ^bc^
	3β-Cholic acid	1.055 ± 0.141 ^cd^	0.756 ± 0.008 ^d^	4.771 ± 0.046 ^a^	1.696 ± 0.419 ^bc^	2.020 ± 0.482 ^b^
	Ursocholic acid	0.450 ± 0.032 ^c^	0.583 ± 0.029 ^c^	6.364 ± 0.761 ^a^	1.313 ± 0.073 ^c^	2.955 ± 0.174 ^b^
	α-Muricholic acid	22.131 ± 2.816 ^d^	24.644 ± 1.165 ^d^	459.425 ± 47.754 ^a^	172.002 ± 7.501 ^b^	103.597 ± 14.512 ^c^
	β-Muricholic acid	73.078 ± 10.759 ^d^	72.437 ± 0.764 ^d^	1341.096 ± 78.128 ^a^	372.051 ± 6.541 ^c^	727.832 ± 73.249 ^b^
	ω-Muricholic acid	209.451 ± 3.179 ^bc^	262.222 ± 6.943 ^b^	646.883 ± 30.113 ^a^	145.387 ± 1.774 ^c^	207.761 ± 47.591 ^bc^
Primary conjugated bile acids	Glycocholic acid	0.025 ± 0.004 ^d^	0.022 ± 0.001 ^d^	0.099 ± 0.019 ^c^	0.381 ± 0.031 ^a^	0.282 ± 0.027 ^b^
	Glycochenodeoxycholic acid	0.002 ± 0 ^b^	0.001 ± 0 ^b^	0.017 ± 0.001 ^b^	0.006 ± 0 ^b^	0.063 ± 0.030 ^a^
	Taurocholic acid	2.533 ± 0.146 ^c^	4.044 ± 0.086 ^b^	6.184 ± 0.524 ^a^	6.053 ± 0.219 ^a^	4.195 ± 0.867 ^b^
	Taurochenodeoxycholic acid	0.241 ± 0.026 ^c^	0.251 ± 0.032 ^bc^	1.784 ± 0.333 ^a^	1.391 ± 0.262 ^abc^	1.502 ± 0.951 ^ab^
	Glycohyocholic acid	0.001 ± 0 ^b^	0.001 ± 0 ^b^	0.008 ± 0.002 ^a^	0.005 ± 0.001 ^ab^	0.007 ± 0.002 ^a^
	Taurohyocholic acid	0.028 ± 0.007 ^c^	0.013 ± 0.003 ^c^	0.213 ± 0.064 ^a^	0.033 ± 0.008 ^bc^	0.113 ± 0.009 ^b^
	Tauro-α-muricholic acid	1.163 ± 0.248 ^c^	1.066 ± 0.291 ^c^	11.659 ± 1.063 ^a^	4.137 ± 0.665 ^b^	5.833 ± 0.931 ^b^
	Tauro-β-muricholic acid	6.393 ± 0.459 ^c^	4.521 ± 0.432 ^c^	38.594 ± 2.579 ^a^	10.763 ± 1.468 ^c^	26.418 ± 5.857 ^b^
	Tauro-ω-muricholic acid	3.232 ± 0.099 ^d^	3.636 ± 0.081 ^cd^	17.963 ± 2.906 ^a^	7.365 ± 0.697 ^c^	11.994 ± 1.132 ^b^
	Taurohyodeoxycholic acid	0.217 ± 0.016 ^b^	0.173 ± 0.046 ^b^	0.681 ± 0.144 ^a^	0.313 ± 0.073 ^ab^	0.612 ± 0.328 ^ab^
Secondary free bile acids	Deoxycholic acid	158.811 ± 51.001 ^d^	256.597 ± 7.119 ^c^	516.194 ± 45.501 ^a^	327.050 ± 31.743 ^bc^	351.762 ± 5.591 ^b^
	Lithocholic acid	37.352 ± 1.625 ^c^	25.654 ± 1.998 ^c^	100.059 ± 5.568 ^a^	74.532 ± 1.026 ^b^	65.227 ± 12.172 ^b^
	Ursodeoxycholic acid	25.641 ± 2.001 ^c^	31.756 ± 5.668 ^bc^	103.881 ± 16.002 ^a^	57.282 ± 3.153 ^b^	52.882 ± 14.702 ^bc^
	3β-Ursodeoxycholic acid	1.388 ± 0.261 ^b^	1.491 ± 0.277 ^b^	5.477 ± 0.081 ^a^	4.929 ± 0.471 ^a^	5.921 ± 0.821 ^a^
	Isolithocholic acid	9.479 ± 0.982 ^bc^	6.751 ± 0.718 ^c^	19.971 ± 3.308 ^a^	14.202 ± 2.218 ^b^	12.054 ± 1.579 ^bc^
	Isodeoxycholic acid	0 ± 0	0 ± 0	0 ± 0	0 ± 0	0 ± 0
	Murideoxycholic acid	3.697 ± 0.537 ^c^	2.721 ± 0.245 ^c^	17.713 ± 4.071 ^a^	8.643 ± 3.499 ^bc^	12.419 ± 1.668 ^ab^
	23-Nordeoxycholic acid	0.023 ± 0.002 ^c^	0.125 ± 0.036 ^a^	0.088 ± 0.016 ^ab^	0.042 ± 0.005 ^bc^	0.032 ± 0.021 ^c^
	7-ketoLithocholic acid	0.221 ± 0.049 ^c^	0.229 ± 0.022 ^c^	1.492 ± 0.141 ^a^	0.958 ± 0.074 ^b^	1.277 ± 0.211 ^ab^
	12-ketolithocholic acid	19.256 ± 3.832 ^b^	25.956 ± 0.251 ^b^	72.017 ± 23.181 ^a^	34.653 ± 3.965 ^b^	42.802 ± 0.841 ^b^
	7-ketodeoxycholic acid	2.703 ± 0.413 ^c^	4.106 ± 0.325 ^c^	16.065 ± 3.024 ^a^	9.499 ± 1.203 ^b^	16.489 ± 2.655 ^a^
	12-ketochenodeoxycholicacid	0 ± 0	0 ± 0	0 ± 0	0 ± 0	0 ± 0
	7,12-Diketolithocholic acid	0.054 ± 0.009 ^b^	0.024 ± 0.009 ^b^	0.182 ± 0.009 ^ab^	0.098 ± 0.009 ^b^	0.364 ± 0.211 ^a^
	3-Dehydrocholic acid	0.195 ± 0.025 ^c^	0.256 ± 0.079 ^c^	2.061 ± 0.021 ^ab^	1.502 ± 0.182 ^bc^	3.481 ± 1.305 ^a^
	Dehydrocholic acid	0 ± 0	0 ± 0	0 ± 0	0 ± 0	0 ± 0
	Dehydrolithocholic acid	4.209 ± 1.152 ^ab^	1.395 ± 0.056 ^b^	5.529 ± 1.855 ^a^	1.678 ± 0.237 ^b^	3.639 ± 1.197 ^ab^
Secondary conjugated bile acids	Glycoursodeoxycholic acid	0.004 ± 0 ^c^	0.002 ± 0 ^c^	0.018 ± 0.003 ^b^	0.006 ± 0.002 ^c^	0.049 ± 0.001 ^a^
	Tauroursodeoxycholic acid	0.591 ± 0.067 ^c^	0.656 ± 0.213 ^c^	1.861 ± 0.013 ^b^	0.871 ± 0.095 ^c^	2.821 ± 0.390 ^a^
	Glycodeoxycholic acid	0.394 ± 0.005 ^b^	0.221 ± 0.003 ^b^	1.634 ± 0.461 ^a^	0.472 ± 0.168 ^b^	1.221 ± 0.066 ^a^
	Glycolithocholic acid	0.046 ± 0.010 ^bc^	0.018 ± 0.006 ^c^	0.131 ± 0.026 ^ab^	0.069 ± 0.006 ^bc^	0.211 ± 0.069 ^a^
	Taurolithocholic acid	0.118 ± 0.022 ^c^	0.109 ± 0.005 ^c^	0.810 ± 0.085 ^b^	0.753 ± 0.116 ^b^	1.434 ± 0.173 ^a^
	Taurodeoxycholate acid	1.009 ± 0.004 ^b^	1.661 ± 0.232 ^b^	6.598 ± 0.503 ^a^	5.758 ± 0.515 ^a^	6.676 ± 0.611 ^a^
	Lithocholic acid 3-sulfate	0.005 ± 0.001 ^ab^	0.002 ± 0 ^b^	0.009 ± 0.004 ^a^	0.007 ± 0.002 ^ab^	0.006 ± 0 ^ab^
	Chenodeoxycholic acid-3-β-D-glucuronide	0.033 ± 0.003 ^c^	0.055 ± 0.006 ^bc^	0.251 ± 0.034 ^a^	0.113 ± 0.012 ^b^	0.131 ± 0.053 ^b^
	Chenodeoxycholic Acid 24-Acyl-β-D-glucuronide	0 ± 0	0 ± 0	0 ± 0	0 ± 0	0 ± 0
Primary free bile acids		346.721 ± 13.458 ^d^	401.315 ± 6.365 ^d^	2776.465 ± 70.256 ^a^	798.456 ± 25.235 ^c^	1282.635 ± 166.455 ^b^
Primary conjugated bile acids		13.838 ± 1.009 ^d^	13.733 ± 0.638 ^d^	77.207 ± 3.465 ^a^	30.451 ± 0.856 ^c^	51.025 ± 10.131 ^b^
Secondary free bile acids		263.033 ± 59.536 ^d^	357.066 ± 4.689 ^c^	860.735 ± 3.379 ^a^	535.071 ± 23.653 ^b^	568.356 ± 26.533 ^b^
Secondary conjugated bile acids		2.203 ± 0.094 ^c^	2.727 ± 0.010 ^c^	11.314 ± 0.098 ^a^	8.052 ± 0.889 ^b^	12.551 ± 1.363 ^a^
Total primary bile acids		360.559 ± 14.467 ^d^	415.049 ± 7.004 ^d^	2853.672 ± 73.721 ^a^	828.908 ± 26.092 ^c^	1333.661 ± 176.587 ^b^
Total secondary bile acids		265.236 ± 59.631 ^d^	359.794 ± 4.701 ^c^	872.051 ± 3.281 ^a^	543.124 ± 24.543 ^b^	580.907 ± 27.896 ^b^
Total bile acids		625.795 ± 74.098 ^d^	774.843 ± 2.304 ^d^	3725.723 ± 77.002 ^a^	1372.032 ± 50.636 ^c^	1914.568 ± 204.484 ^b^
Primary/secondary bile acids ratio		1.399 ± 0.266 ^c^	1.153 ± 0.034 ^c^	3.272 ± 0.072 ^a^	1.526 ± 0.020 ^c^	2.289 ± 0.194 ^b^
Conjugated/free bile acids ratio		0.026 ± 0.001 ^bc^	0.021 ± 0 ^d^	0.024 ± 0 ^cd^	0.028 ± 0 ^b^	0.034 ± 0.002 ^a^

Data are presented as mean ± standard deviation. *n* = 6. Different lowercase letters (a, b, c, d) indicate significant differences among groups for the same bile acid (*p* < 0.05).

## Data Availability

The original contributions presented in this study are included in the article/Supplementary Materials. Further inquiries can be directed to the corresponding author.

## Supplementary Materials

The following supporting information can be downloaded at https://www.mdpi.com/article/10.3390/nu18071105/s1, Table S1. Feed composition and energy-supplying ratio; Table S2. Composition of the extracts.

## Abbreviations

The following abbreviations are used in this manuscript:

NAFLD	Non-alcoholic fatty liver disease
HFD	High-fat diet
PLE	Pepper leaf extracts
SE	Spinach extracts
OCA	Obeticholic acid
SCFA	Short-chain fatty acid
LPS	Lipopolysaccharide
FXR	Farnesoid X receptor
H&E	Hematoxylin-eosin
TC	Total cholesterol
TG	Triglyceride
DAO	Diamine oxidase
LC-MS	Liquid chromatography-mass spectrometry
SPF	Specific pathogen-free
CON	Control group
CON_PLE	Control + pepper leaf extracts group
HFD_PLE	High-fat diet + pepper leaf extracts group
HFD_SE	High-fat diet + spinach extracts group
HFD_OCA	High-fat diet + obeticholic acid group
CMC-Na	Carboxymethyl cellulose
eWAT	Epididymal white adipose tissue
iWAT	Inguinal white adipose tissue
pWAT	Perirenal white adipose tissue
sBAT	Scapular brown adipose tissue
ASVs	Amplicon sequence variants
KEGG	Kyoto Encyclopedia of Genes and Genomes
FER	Food efficiency ratio
PAS	Periodic acid-Schiff
PCoA	Principal coordinates analysis
PLS-DA	Partial Least Squares-Discriminant Analysis
WAT	White adipose tissue
