# Supplementary material for: Pepper Leaf Extracts Alleviate HFD-Induced Metabolic Disorders via Microbiota-Driven Intestinal Barrier Repair and Bile Acid Reprogramming
Source: Nutrients. 2026 Mar 30;18(7):1105. doi: 10.3390/nu18071105 (PMC13075201; doi:10.3390/nu18071105)
Supplement: Supplementary file 1 [file nutrients-18-01105-s001.zip › nutrients-4102507-supplementary.pdf]

Supplementary Table S1. Feed composition and energy-supplying ratio.

Ingredient	XT304 (kcal %)	XT310 (kcal %)
Casein	19.82	19.82
L-Cystine	0.30	0.30
Corn starch	34.68	0.00
Fructose	0.00	19.82
Maltodextrin	8.42	9.91
Sucrose	9.91	9.91
Glucose	16.75	0.00
Cellulose	0.00	0.00
Soybean oil	5.57	5.57
Lard	4.46	4.46
Palm oil	0.00	30.10
Mineral mix S10026B	0.00	0.00
Vitamin mix V10001C	0.10	0.10
Choline bitartrate	0.00	0.00
Cholesterol	0.00	0.00
Energy（kcal/g）	4.04	4.04

Supplementary Table S2. Composition of the extracts.

Ingredient	Pepper leaf extracts	Spinach extracts
	g/100g (dry basis, DB)	g/100g (dry basis, DB)
Total Carbohydrates	3.92	3.63
Crude Protein	4.30	4.13
Crude Fat	74.68	75.13
Total Chlorophyll	3.96	4.01
